# AMPKα1 deletion in myofibroblasts exacerbates post-myocardial infarction fibrosis by a connexin 43 mechanism

**DOI:** 10.1007/s00395-021-00846-y

**Published:** 2021-02-09

**Authors:** Cécile Dufeys, Evangelos-Panagiotis Daskalopoulos, Diego Castanares-Zapatero, Simon J. Conway, Audrey Ginion, Caroline Bouzin, Jérôme Ambroise, Bertrand Bearzatto, Jean-Luc Gala, Stephane Heymans, Anna-Pia Papageorgiou, Stefan Vinckier, Julien Cumps, Jean-Luc Balligand, Maarten Vanhaverbeke, Peter Sinnaeve, Stefan Janssens, Luc Bertrand, Christophe Beauloye, Sandrine Horman

**Affiliations:** 1 Pôle de Recherche Cardiovasculaire (CARD), Institut de Recherche Expérimentale et Clinique (IREC), Université Catholique de Louvain (UCLouvain), 55, Avenue Hippocrate, 1200 Brussels, Belgium; 2 HB Wells Center for Pediatric Research, Indiana University School of Medicine, Indianapolis, IN USA; 3 IREC Imaging Platform, Institut de Recherche Expérimentale et Clinique (IREC), Université Catholique de Louvain (UCLouvain), Brussels, Belgium; 4 Centre de Technologies Moléculaires Appliquées, Institut de Recherche Expérimentale et Clinique, UCL, Brussels, Belgium; 5 Center for Heart Failure Research, Cardiovascular Research Institute Maastricht (CARIM), Maastricht University, Maastricht, The Netherlands; 6 Department of Cardiovascular Sciences, KU Leuven, Louvain, Belgium; 7 Center for Cancer Biology, University of Leuven and VIB, Louvain, Belgium; 8 Pôle de Pharmacologie et de Thérapeutique (FATH), Institut de Recherche Expérimentale et Clinique (IREC), Université Catholique de Louvain (UCLouvain), Brussels, Belgium; 9 Department of Cardiovascular Medicine, Leuven University Hospitals, Louvain, Belgium; 10 Division of Cardiology, Cliniques Universitaires Saint-Luc, Brussels, Belgium

**Keywords:** Cardiac fibrosis, Cardiac fibroblast, Myofibroblast, AMPKα1, Connexin 43, miR-125b-5p

## Abstract

We have previously demonstrated that systemic AMP-activated protein kinase α1 (AMPKα1) invalidation enhanced adverse LV remodelling by increasing fibroblast proliferation, while myodifferentiation and scar maturation were impaired. We thus hypothesised that fibroblastic AMPKα1 was a key signalling element in regulating fibrosis in the infarcted myocardium and an attractive target for therapeutic intervention. The present study investigates the effects of myofibroblast (MF)-specific deletion of AMPKα1 on left ventricular (LV) adaptation following myocardial infarction (MI), and the underlying molecular mechanisms. MF-restricted AMPKα1 conditional knockout (cKO) mice were subjected to permanent ligation of the left anterior descending coronary artery. cKO hearts exhibit exacerbated post-MI adverse LV remodelling and are characterised by exaggerated fibrotic response, compared to wild-type (WT) hearts. Cardiac fibroblast proliferation and MF content significantly increase in cKO infarcted hearts, coincident with a significant reduction of connexin 43 (Cx43) expression in MFs. Mechanistically, AMPKα1 influences Cx43 expression by both a transcriptional and a post-transcriptional mechanism involving miR-125b-5p. Collectively, our data demonstrate that MF-AMPKα1 functions as a master regulator of cardiac fibrosis and remodelling and might constitute a novel potential target for pharmacological anti-fibrotic applications.

**Supplementary Information:**

The online version contains supplementary material available at 10.1007/s00395-021-00846-y.

## Introduction

Myocardial infarction (MI) is a major cause of mortality and morbidity worldwide, in spite of improved prevention and therapy [23, 24]. The acute loss of cardiomyocytes is followed by adverse cardiac remodelling because of excessive volume and pressure load on non-infarcted areas. This process encompasses the development of cardiomyocyte hypertrophy, myocardial fibrosis, dilatation, and electrophysiological changes, all leading to left ventricular (LV) dysfunction, and eventually, heart failure and death [18, 24]. Fibrosis holds a prominent role in adverse LV remodelling, and has become a prime focus in cardiac research [17].

Resident cardiac fibroblasts (CFs) are present in the myocardium, playing a key role in regulating extracellular matrix (ECM) turnover [65]. Under stress conditions, as in MI, CFs become activated and respond to changes in their microenvironment by acquiring a wide range of phenotypic profiles [19, 26]. They notably differentiate into myofibroblasts (MFs), with enhanced ECM synthesis and smooth muscle-like contractile properties [19]. Whereas MFs can arise from a variety of cell types, most originate from resident CFs [31, 46]. MFs express α-smooth muscle actin (αSMA) and orchestrate the deposition of collagen and secretion of a wide range of structural and non-structural ECM proteins leading to the formation of a stable scar [19]. As the scar matures, the MFs cease to express αSMA and achieve a new differentiated state (matrifibrocyte), which is characterised by high expression of cartilage and tendon genes [19, 26]. Although the fibrotic response proves essential to scar formation and survival in the early post-MI stages, it eventually leads to deleterious adverse LV remodelling in the longer term, both in the infarcted and the remote myocardium [17].

Fibrosis is amenable to numerous regulatory mechanisms, notably by Transforming growth factor (TGF)-β signalling [33]. Among less frequently studied pathways, connexins (Cxs) can likewise trigger fibrosis [15, 16, 29]. Cxs are membrane proteins that assemble into hexamers and form hemichannels or gap junction channels with essential roles in the communication with the extracellular space or between adjacent cells. Several Cxs exist, of which Cx43 is the most abundantly expressed isoform in cardiac ventricles [44]. Cx43 is not only a key element in propagating the action potential (myocyte-myocyte communication), but it also facilitates the communication between myocytes and CFs [41, 58]. Interestingly, in the atrial fibrillation disease process, atrial gap junction interruption, due to Cx43 downregulation, is associated with enhanced fibrosis, which contributes further to the slowing of conduction and formation of reentry [48]. This characteristic structural and electrical remodelling in atria can be recapitulated in cardiac-specific liver kinase B1 (LKB1) knockout (KO) mice, a model that accurately reproduces human atrial fibrillation [52]. LKB1 encodes a serine/threonine protein kinase which functions upstream of the AMP-activated protein kinase (AMPK) superfamily. Several recent studies support the link between AMPK signalling and Cx43 expression, not only in cardiomyocytes [1, 40] but also in CFs [7], proposing a role for this pathway in the fibrotic response commonly seen with a variety of cardiac pathologies.

AMPK, a highly conserved enzyme, is omnipresent in all eukaryotes. The AMPK subunits (α, β, and γ) demonstrate tissue-specific expression with AMPKα2 being the most commonly expressed α subunit in murine cardiomyocytes. Our group, as well as others, have demonstrated its critical protective role in post-ischaemic and failing hearts [11], against excessive cardiac hypertrophy [20, 73, 74], and arrhythmias [4]. On the other hand, AMPKα1 is principally expressed in non-myocyte cardiac cells, including CFs [49], endothelial cells [5], smooth muscle cells [25], platelets [38, 50], and mesenchymal stem cells [12]. We have previously reported on cardiac AMPKα1 as a crucial player in regulating the CFs/MFs ratio following ischaemia, thereby demonstrating its cardinal significance in limiting LV remodelling post-MI [49]. However, the exact contribution of MF-AMPKα1 towards this cardioprotective effect is still unclear.

In the current study, we have achieved MF-specific AMPKα1 deletion using Cre-recombinase driven by the periostin (Postn) promoter in AMPKα1^fl/fl^ mice. We report that MF-specific knockout (cKO) of AMPKα1 was associated with enhanced fibrosis, as well as adverse cardiac remodelling following MI. Our data shed light onto the intricate roles of myofibroblastic AMPKα1, underscoring the role of Cx43 as a novel target involved in adverse LV remodelling post-MI.

## Methods

A detailed description of reagents and methods is also presented in the Electronic Supplementary Material.

### Animal care

Animal handling and experimental procedures were approved by the local authorities (Comité d’éthique facultaire pour l’expérimentation animale, 2012/UCL/MD/003 and 2016/UCL/MD/027) and performed in accordance with the Guide for the Care and Use of Laboratory Animals, published by the US National Institutes of Health (NIH Publication, revised 2011). All animals were housed with a 12-h/12-h light/dark cycle, with the dark cycle occurring from 6.00 p.m. to 6.00 a.m. Mice were observed daily with free access to water and standard chow.

### Experimental animals

We generated an injury-inducible MF-specific KO mouse model in which AMPKα1 could be specifically deleted in MFs in response to cardiac injury. In this model, the Cre-recombinase is driven by a 3.9-kb partial mouse Postn promoter (kindly provided by Simon J. Conway, Indiana University) [35, 37, 60, 64]. Postn-Cre^+/−^ mice were bred with AMPKα1^fl/fl^ mice that possess loxP sites flanking exon 3 of PRKAA1 gene (commercially available, Jackson Laboratory, #014141) to generate MF-specific AMPKα1 KO mice (cKO). Postn-Cre^−/−^; AMPKα1^fl/fl^ (WT) littermates were used as controls. cKO progeny were viable, fertile and reproduced at expected Mendelian ratios, and showed no overt pathological phenotypes.

To further demonstrate the MF-specific activity of Cre-recombinase, we used a reporter mice model. Postn-Cre mice were bred with R26-stop^fl/fl^-enhanced yellow fluorescent protein (EYFP) reporter animals exhibiting the EYFP gene into the Rosa26 locus, preceded by a loxP-flanked stop sequence (kindly provided by Patrick Gilon, UCLouvain) [63].

### Mouse model of myocardial infarction

MI was induced in 10- to 12-week-old male and female mice by left anterior descending (LAD) coronary artery permanent ligation performed by an experienced operator. Animals were pre-treated with buprenorphine hydrochloride (0.1 mg/Kg, i.p.), anesthetised with 2,2,2-tribromoethanol/2-methyl-2-butanol (310 mg/Kg, i.p.), and their tracheas were intubated. After left-sided thoracotomy at the fourth intercostal space, the LAD coronary artery was ligated below its origin using 8.0 polyamide silk 1–2 mm. Infarction was confirmed by visualisation of ventricle cyanosis under the microscope. The heart was subsequently replaced into the thoracic cavity, and the chest closed. Sham animals underwent a similar surgical procedure, except for the coronary ligation. Animals were euthanized with pentobarbital sodium (90 mg/Kg, i.p.) on days 3, 7, 14, or 3 months post-surgery. Mice were randomly assigned to sham or MI. All investigators conducting the experiments, acquiring and analysing the data were blinded to the experimental groups.

### Echocardiography

Transthoracic echocardiography was performed on anesthetised mice (3–4% of isoflurane for induction and 1–2% for maintenance, in 100% oxygen) using a Vevo 2100 Imaging System (FUJIFILM VisualSonics, Toronto, Canada) equipped with a 30 MHz transducer. Cardiac parameters were measured in 2-dimensional (2D) B-mode long axis and M-mode. Infarct extent was determined by analysing regional LV wall motion 2 days post-MI, based on adjustments for human heart imaging [6]. Echocardiography was performed at baseline (1–3 days pre-op), 1, 7 and 14 days, and 1, 2, and 3 months post-MI. LV volumes were measured using B-mode parasternal long-axis view, at end-systole and end-diastole, from which ejection fraction (EF %) was deduced. LV mass was calculated from LV long-axis measurements and normalised by tibia length. Fractional shortening (FS %) was calculated using internal LV dimensions and wall thickness (septum and posterior wall) measurements at end-systole and end-diastole obtained from M-mode recordings. All measurements and analysis were performed by the same experienced operator who was blinded to the experimental groups.

### Picrosirius red staining and polarised light microscopy

Heart sections were treated with 1% phosphomolybdic acid for 2 min, washed with H_2_O and incubated in 0.1% picrosirius red solution for 2 h at room temperature. Sections were then quickly rinsed in hydrochloric acid 0.01 M, washed with H_2_O, and mounted with Entellan. Stainings were digitised using the Leica SCN400 slide scanner (Leica Microsystems). Myocardial fibrosis was analysed on four to seven heart sections per animal using TissueIA software (Leica Biosystems). Fibrosis was quantified as picrosirius red positive area relative to total area of interest. Scar circumference was assessed by measuring the largest circumference of serial sections from apex to base. Collagen fibres quality was investigated by analysing picrosirius red staining using Axioskop 40 microscope (Carl Zeiss) with a polarising filter. The amount of thick, closely packed, red–orange collagen fibres was compared with that of thin, loosely assembled, green–yellow fibres. Analysis was conducted using FRIDA software (Framework for Image Dataset Analysis, The Johns Hopkins University, USA).

### Fluorescent immunohistochemistry

After endogenous peroxidases inhibition with 3% H_2_O_2_ for 20 min and antigen retrieval by microwave heating for 15 min in citrate buffer pH 6, heart sections were permeabilized in Tris-Buffered Saline (TBS)/0.1% Tween 20 and blocked for 30 min with 5% BSA solution. EYFP expression, CF myodifferentiation and proliferation were assessed by double staining with GFP (1:2000) αSMA (1:5000) or PCNA (1:800), respectively, as well as vimentin (1:10,000) antibodies. Primary antibodies were incubated for 90 min at room temperature, with slides washed with TBS/0.1% Tween 20. Slides were then incubated for 40 min with horseradish peroxidase (HRP)-conjugated polymer secondary antibody at room temperature, washed, and incubated with either Alexa Fluor 488 or 555 tyramide reagent (1:200) for 10 min. After washing, nuclei were stained with Hoechst 33,342 (10 µg/mL), and slides were mounted. Omission of the primary antibody served as negative control. Pictures were acquired using an Axio Imager.z1 microscope (Carl Zeiss) with a 20× objective. EYFP expression, myodifferentiation and proliferation were analysed along the entire infarcted LV wall, remote and border areas, on 4 randomly selected heart sections per animal (10–20 images per animal), using Axiovision software (Carl Zeiss).

### Electron microscopy

Hearts were rinsed using retrograde perfusion with 0.9% NaCl, followed by perfusion with Karnovsky fixative. The heart was separated into infarct area, border zone, and remote area, and cut into 1 mm^3^ pieces. Following overnight incubation in Karnovsky fixative, heart pieces were washed and transferred to a 1% osmium tetroxide postfixative solution buffered with sodium cacodylate buffer. Prior to embedding in Epon, the material was dehydrated in a graded ethanol series. Semi-thin (± 1 mm) sections were cut with a Reichert Jung Ultracut E microtome and stained with 0.1% thionin–0.1% methylene blue. The ultra-thin (± 70 nm) sections, on copper grids, were stained with uranyl acetate and lead citrate. Transmission electron microscopy was performed on a JEOL JEM1400 (JEOL Europe BV) (VIB Bio Imaging Core, Leuven Platform). Both collagen fibre density and diameter were determined using Image J software (version 1.6, National Institutes of Health, Bethesda, USA).

### Isolation of adult murine non-myocyte cells from cardiac muscle tissue

LVs were excised, rinsed in cold PBS, minced in small pieces, and digested using 100 U/mL Type II collagenase for 15 min at 37 °C. The collagenase medium containing the cardiac cell suspension was centrifuged 5 min at 1000*g*, and cells were resuspended in Dulbecco’s modified eagle medium (DMEM) using 10% FBS and 2% penicillin/streptomycin. The digestion step was repeated until all heart pieces were digested (around 6–7 times). Cells were plated and allowed to attach for 1 h, following which the medium was changed to remove poorly adherent cells including cardiomyocytes, endothelial cells, and blood cells.

### Primary human cardiac fibroblast culture and treatments

Human CFs (HCFs) were purchased from ScienCell Research Laboratories (#6300). Cells are isolated from human healthy heart, cryopreserved at passage one and delivered frozen. Each vial contains > 5 × 10^5^ cells in 1 mL volume. They are characterised by immunofluorescence with an antibody specific to fibronectin. HCF are negative for HIV-1, HBV, HCV, mycoplasma, bacteria, yeast, and fungi and guaranteed to further expand for 15 population doublings under the conditions provided by ScienCell Research Laboratories. Cells were cultured according to the manufacturer’s recommendations. They were transfected with 30 nM AMPKα1- or 15 nM Cx43-targeting siRNA constructs or control siRNA (si-scramble) construct using Lipofectamine RNAiMAX transfection reagent for 48 h following the manufacturer’s instructions.

### Cell proliferation assay

Transfected HCFs were serum starved for 24 h prior to stimulation with 1% FBS for 48 h. Gap19 100 µM was added at the time of proliferation stimulation. Proliferation was measured by flow cytometry (BD FACS Calibur, Becton Dickinson) with Click-iT Edu flow cytometry assay kit according to the manufacturer’s recommendations. The data were analysed by FLOWJO software (version 8.8.7, Tree Star and Leland Stanford Junior University).

### Overexpression or knockdown of miR-125b-5p

HCFs were transfected with 40 nM miR-125b-5p mimic (or si-scramble as negative control) for 48 h using Lipofectamine RNAiMAX transfection reagent, following the manufacturer’s instructions. For the endogenous miR-125b-5p knockdown experiment, HCFs were transfected with 30 nM AMPKα1-targeting siRNA or a scramble siRNA for 6 h, as previously described. The medium was then replaced, and HCFs were transfected with 50 nM antagomir-125b-5p or antagomir control for 42 h, using Lipofectamine RNAiMAX transfection reagent.

### mRNA and microRNA extraction and expression analysis

Total RNA was isolated from HCFs, isolated mouse CFs or mouse LV with RNeasy mini-kit and miRNeasy mini-kit, for mRNA and microRNA analyses, respectively, and samples were treated with DNase according to the manufacturer’s instructions. RNA was quantified using NanoDrop (Thermo Fisher Scientific), 1 µg was reverse transcribed, and real-time quantitative PCR (RT-qPCR) using SYBR Green was employed for mRNA and microRNA expression analyses. Reactions were performed on an IQ5 apparatus (Bio-Rad). RPL32 was applied as housekeeping gene for mRNA measurement, and SNORD96A for microRNAs.

### RNA sequencing

Total RNA was isolated from HCFs and treated with DNase as described above. RNA was quantified using Qubit 4 Fluorometer (Thermo Fisher Scientific). RNA integrity was evaluated on the Agilent 2100 Bioanalyzer. All samples had RNA integrity number values ≥ 9.9. Libraries were prepared starting from 150 ng of total RNA using the KAPA RNA HyperPrep Kit with RiboErase following the manufacturer’s recommendations. Libraries were equimolarly pooled and sequenced on a single lane on an Illumina NovaSeq 6000 platform. All libraries were paired-end sequenced and a minimum of 25 million of paired-end reads were generated per sample. All sequencing data were analysed using the Automated Reproducible MOdular workflow for preprocessing and differential analysis of RNA-seq data (ARMOR) pipeline [51]. In this pipeline, reads underwent a quality check using FastQC (Babraham Bioinformatics). Quantification and quality control results were summarised in a MultiQC report [14] before being mapped using Salmon [53] to the transcriptome index which was built using all Ensembl cDNA sequences obtained in the Homo_sapiens.GRCh38.cdna.all.fa file. Then, estimated transcript abundances from Salmon were imported into R using the tximeta package [61] and analysed for differential gene expression with edgeR [56]. Raw and processed RNA-seq data were deposited and made publicly available on the Gene Expression Omnibus (GSE147470).

### Statistical analysis

Data are expressed as means ± SEM. Normal distribution of continuous variables was tested according to the Shapiro–Wilk method and equality of variances. Statistical significance was evaluated using unpaired student *t* test for comparing two groups, when they had passed the normality test. The non-parametric Mann–Whitney test was applied for comparing two groups having failed the normality test. Either one-way or two-way ANOVA were employed for multiple comparisons. Post hoc pairwise comparisons were carried out using the Sidak method. Survival analysis was conducted by means of the log-rank test. Correlations were analysed using the Pearson coefficient *r* calculation. Data were analysed using GraphPad prism 8 statistical software.

## Results

### Generation and characterisation of MF-specific AMPKα1 KO mice

**Fig. 1:**
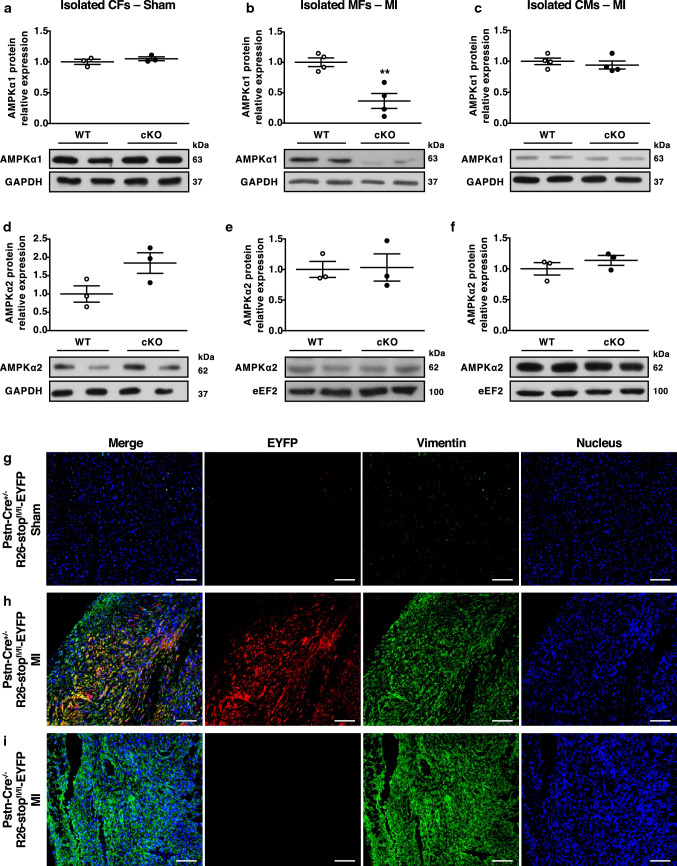
Generation and characterisation of MF-specific AMPKα1 KO mice. **a**–**f** Cardiac fibroblasts (CFs) (**a**, **d**), myofibroblasts (MFs) (**b**, **e**) and cardiomyocytes (CMs) (**c**, **f**) were isolated from MF-specific AMPKα1 KO (cKO) and littermates WT (WT) mouse hearts 14 days after sham (**a**, **d**) or myocardial infarction (MI) (**b**, **c**, **e**, **f**) surgery. Western blotting was performed for analysing AMPKα1 (**a**–**c**) or AMPKα2 (**d**–**f**) expression (*n* = 3–4 mice/group). Dot plots represent data from individual mouse hearts, as well as mean ± SEM. Statistical significance was determined by unpaired *t* test. **p < 0.01 compared to WT. **g**–**i** Postn-Cre^+/−^;Rosa26-STOP^fl/fl^-enhanced yellow fluorescent protein (EYFP) (**g**, **h**) and Postn-Cre^−/−^;Rosa26-STOP^fl/fl^-EYFP (**i**) mouse hearts were collected 7 days following MI (**h**, **i**) or sham surgery (**g**), with EYFP expression assessed by immunohistofluorescence. Representative images of EYFP staining. Magnification: ×10. Scale bars: 100 µm

To evaluate MF AMPKα1’s role in regulating MI-induced fibrotic remodelling, we generated mice in which AMPKα1 gene function was specifically deleted in MFs. In this model, Cre-recombinase is driven by a partial mouse Postn promoter [35, 37, 60, 64]. While Postn expression is negligible in the healthy adult heart, it significantly and specifically increases in MFs in the first few days following injury [31]. Importantly for our study, Postn expression is only induced in MF lineage following cardiac insult, and is not present in either resident CFs or myocytes [31, 64]. To generate injury-inducible MF-specific AMPKα1 KO mice (cKO), AMPKα1^fl/fl^ mice were bred with Postn-Cre^+/−^ mice. Postn-Cre^−/−^;AMPKα1^fl/fl^ (WT) littermates were employed as controls. To investigate the Cre-mediated AMPKα1 deletion’s extent and specificity, we removed the left ventricle from these mice at 14 days post-surgery, and isolated interstitial non-myocyte cells and cardiomyocytes for culture. Non-myocyte cells isolated from sham-operated hearts generally did not express αSMA (Suppl. Figure 1a). Nonetheless, non-myocyte cells isolated from infarcted hearts all became αSMA-positive, displaying characteristic stress fibres of MFs (Suppl. Figure 1b). AMPKα1 expression levels were comparable in CFs isolated from sham-operated mice of both genotypes (Fig. 1a). After MI, AMPKα1 levels dramatically decreased in cKO, in comparison to WT MFs (Fig. 1b), while remaining unchanged in cardiomyocytes (Fig. 1c). AMPKα2 expression did not differ after sham or MI surgery, either in CFs/MFs (Fig. 1d, e, respectively) or cardiomyocytes (Fig. 1f) isolated from WT and cKO hearts. To further demonstrate the non-myocyte specific activity of Cre-recombinase in our animal model, Postn-Cre mice were bred with R26-stop^fl/fl^-EYFP reporter animals exhibiting the enhanced yellow fluorescent protein (EYFP) gene into the Rosa26 locus, preceded by a loxP-flanked stop sequence [63]. At the histological level, sham-operated Postn-Cre^+/−^;R26-stop^fl/fl^-EYFP mice displayed very low or no EYFP + interstitial cell levels in the heart (Fig. 1g); whereas 7 days post-MI injury, these mice had abundant EYFP staining, colocalizing with vimentin in the non-myocyte cells within the infarcted area (Fig. 1h). EYFP expression was low or undetectable in MI-operated Postn-Cre^−/−^;R26-stop^fl/fl^-EYFP hearts (Fig. 1i).

### MF-specific AMPKα1 deficiency aggravates LV adverse remodelling post-MI

**Fig. 2:**
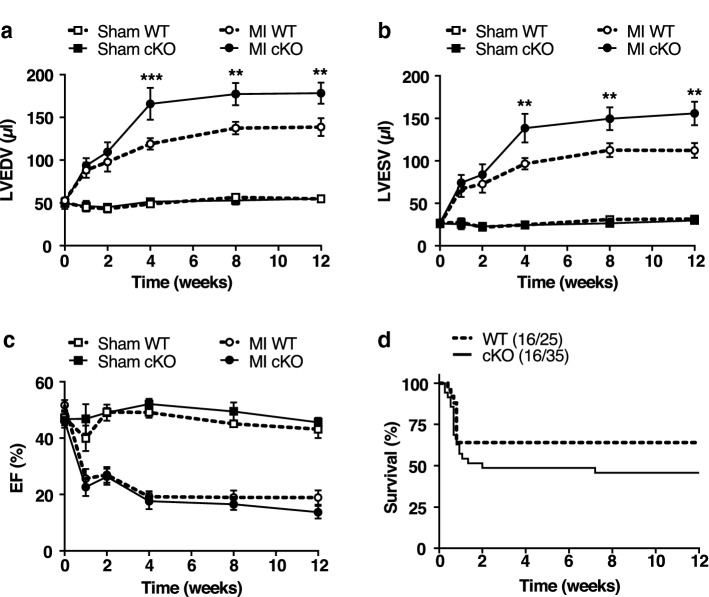
MF-specific AMPKα1 deficiency aggravates LV adverse remodelling post-MI. **a**–**c** Left ventricular end-diastolic (LVEDV) (**a**) and -systolic (LVESV) (**b**) volumes and ejection fraction (EF) (**c**) of MF-specific AMPKα1 KO (cKO) and littermates WT (WT) mice measured by transthoracic echocardiography at the indicated time points post-myocardial infarction (MI) or sham surgery (*n* = 10–11 mice/group). Results are expressed as mean ± SEM. Statistical significance was determined by two-way ANOVA followed by Sidak’s multiple comparisons test. **p < 0.01, ***p < 0.001 compared to corresponding WT. **d** Kaplan–Meier survival curves of cKO and WT mice until 3 months after MI (*n* = 25–35 mice/group). Differences between groups were determined by the log-rank test, *p* = 0.25

To investigate MF AMPKα1’s role in regulating LV remodelling, we first analysed the infarct magnitude by echocardiographic assessment of regional LV wall motion of each operated mouse at 2 days post-MI [49]. The extent of infarcted myocardium did not differ between cKO and WT mice (WT: 7.56 ± 0.17 vs cKO: 7.71 ± 0.16 number of akinetic segments, *n* = 55/59). At baseline, chamber dimensions and LV function were comparable between the two genotypes (Fig. 2a–c and Table 1). From 1 month until 3 months post-MI, cKO animals exhibited a strong increase in LV end-diastolic (LVEDV) and -systolic (LVESV) volumes in comparison to WT littermates (Fig. 2a, b)*.* It was associated with a significant increase in the LV’s internal dimension (both in diastole and systole), while no clear difference was observed in intraventricular septum or posterior wall thickness (Table 1). LV mass normalised to tibia length was similar between WT and cKO, at 3 months post-MI (Table 1). Consistent with these data, histological analyses of cardiomyocyte cross-sectional area and blood vessel density in the non-infarcted remote myocardium were not different between cKO and WT mice (Suppl. Figure 2a–c). Lastly, the early mortality (< 7 days) (WT: 54.31% vs cKO: 47.47%, *n* = 116/99) and the incidence of cardiac rupture (WT: 12.07% vs cKO: 19.19%, *n* = 116/99) were similar between genotypes, while impaired cardiac function in cKO animals was associated with a trend towards decreased long-term survival (Fig. 2d).

**Table 1:** Related to Fig. 2 echocardiographic measurements of cKO and WT mice at 3 months after myocardial infarction

Variable	Sham 3 months		MI 3 months	
	WT (*n* = 7)	cKO (*n* = 5)	WT (*n* = 11)	cKO (*n* = 10)
M-mode				
End-diastolic interventricular septal thickness (mm)	0.59 ± 0.08	0.56 ± 0.04	0.70 ± 0.07	0.59 ± 0.07
End-systolic interventricular septal thickness (mm)	0.85 ± 0.12	0.78 ± 0.03	0.88 ± 0.10	0.77 ± 0.10
End-diastolic left ventricular internal diameter (mm)	3.84 ± 0.13	4.09 ± 0.18	5.32 ± 0.15***	5.84 ± 0.14***, ^$^
End-systolic left ventricular internal diameter (mm)	2.80 ± 0.09	3.09 ± 0.16	4.63 ± 0.23	*** 5.34 ± 0.22***, ^$^
End-diastolic left ventricular posterior wall thickness (mm)	0.57 ± 0.07	0.52 ± 0.01	0.56 ± 0.05	0.70 ± 0.06
End-systolic left ventricular posterior wall thickness (mm)	0.88 ± 0.08	0.79 ± 0.04	0.75 ± 0.09	0.72 ± 0.05
Fractional shortening (%)	26.78 ± 2.07	24.47 ± 1.46	13.39 ± 2.07***	8.74 ± 1.87***
2D parasternal long axis				
Left ventricular mass (mg)/tibial length (cm)	45.78 ± 1.95	52.06 ± 4.37	62.29 ± 3.67*	68.00 ± 5.22*
Pulse Doppler				
Heart rate (bpm)	435.80 ± 19.04	416.30 ± 11.69	428.80 ± 23.39	451.40 ± 28.18

Cardiac function parameters of MF-specific AMPKα1 KO (cKO) and littermates WT (WT) mice, quantified by transthoracic echocardiography at 3 months after myocardial infarction (MI) or sham surgery (*n* = 5–11 mice/group). Data are reported as mean ± SEM. Statistical significance was determined by two-way ANOVA followed by Sidak’s multiple comparisons test

*p < 0.05, ***p < 0.001 compared to corresponding sham and ^$^*p* < 0.05 compared to WT MI.

### MF-specific AMPKα1 deficiency enhances LV fibrosis post-MI

**Fig. 3:**
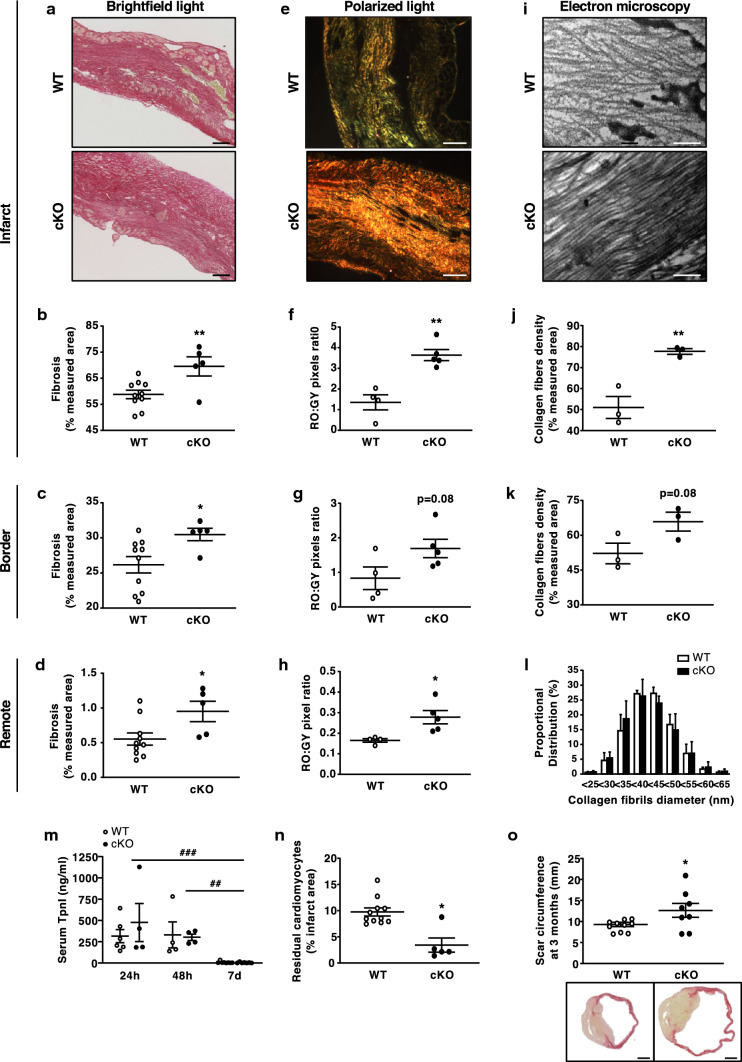
MF-specific AMPKα1 deficiency enhances LV fibrosis post-MI **a** representative images of MF-specific AMPKα1 KO (cKO) and littermates WT (WT) infarct areas stained by picrosirius red and visualised under bright-field light. Magnification: ×10. Scale bars: 100 µm. **b**–**d** Quantifications of fibrosis in the infarct (**b**), border (**c**) and remote (**d**) areas of cKO and WT mice (*n* = 5–10 mice/group). Results are expressed as % of measured tissue area. **e** Representative images of cKO and WT infarct areas stained by picrosirius red and visualised under polarised light. Magnification: ×10. Scale bars: 100 µm. **f**–**h** Quantification of picrosirius red polarised light analysis in the infarct (**f**), border (**g**) and remote (**h**) areas of cKO and WT mice (*n* = 4–5 mice/group). Results are expressed as red–orange on green–yellow pixel ratio in the measured tissue area. **i** Representative images of electron microscopy analysis in the infarct area of cKO and WT mice. Scale bars: 500 nm. **j**–**k** Quantification of collagen fibres density in the extracellular matrix of infarct (**j**) and border (**k**) areas of cKO and WT (*n* = 3 mice/group). Results are expressed as % of measured tissue area. **l** Proportional distribution of collagen fibrils diameter in the infarct area measured by electron microscopy. **m** Serum Troponin I (TpnI) measurement at different time points post-MI in cKO and WT mice. Serum were harvested at 24 h, 48 h and 7 days post-MI and TpnI concentration was measured by ELISA (*n* = 4–8 mice/group). **n** Histological quantifications of residual cardiomyocytes in the infarct of cKO and WT mice (*n* = 5–11 mice/group). Results are expressed as % of measured tissue area. **o** Scar circumference of cKO and WT mice at 3 months post-MI measured from heart sections stained by picrosirius red (*n* = 6–11 mice/group). Scale bars: 1 mm. Hearts were excised at 14 days (**a**–**l**, **n**) and 3 months post-MI. Dot plots represent data from individual mice, as well as mean ± SEM. Statistical significance was determined using unpaired *t* test (**b**–**d**, **f**, **g**, **j**, **k**), Mann–Whitney test (**h**, **n**, **f**), or two-way ANOVA followed by Sidak’s multiple comparisons test (**l**, **m**). *p < 0.05, **p < 0.01 compared to WT and ^##^*p* < 0.01, ^###^*p* < 0.001 compared to corresponding genotype at 7 days post-MI

To investigate MF AMPKα1’s role in cardiac healing, we performed picrosirius red staining at 14 days following MI or sham surgery and measured substantially increased collagen deposition in the infarct, border, and remote areas of cKO mice, compared to WT (Fig. 3a–d). Picrosirius red polarised light microscopy of cKO scars revealed predominant well-aligned and thickly packed collagen fibres, in contrast to WT infarcts (Fig. 3e). Accordingly, the tightly packed versus loosely packed fibres ratio (red–orange:green–yellow) was significantly higher in infarcts, border zones and remote areas of cKO, compared to WT hearts (Fig. 3f–h). Based on ultrastructural analysis with transmission electron microscopy, cKO infarcts were characterised by a denser and more organised collagen matrix (Fig. 3i–k). Nonetheless, collagen fibres diameter did not differ between both genotypes (Fig. 3l), indicating that the cross-linking process was not affected. In support of this, specific AMPKα1 deletion in MFs did not impact lysyl oxidase (LOX) expression in the infarcted myocardium (Suppl. Figure 3a and 3b), despite a trend towards decreased LOX expression in MFs isolated from cKO versus WT hearts (Suppl. Figure 3c). Interestingly, the degree of LV fibrosis was not different between the two genotypes at 3 months following MI, indicating that MF-specific AMPKα1 deficiency merely accelerates fibrotic deposition of collagen in cKO hearts during the early phases of post-infarct healing (Suppl. Figure 4a–h). Importantly, increased fibrosis was not the result of excessive cardiomyocyte mortality in cKO infarcts, as reflected by the measurement of troponin I (TpnI) serum levels at days 1, 2 and 7 post-MI (Fig. 3m). Cardiomyocyte death similarly occurred in the first 2 days in WT and cKO mice, and was barely detected after 7 days (Fig. 3m). Of note, 2 weeks after MI, the number of residual cardiomyocytes in the infarcted myocardium was lower in cKO infarcts (Fig. 3n), possibly as a consequence of increased fibrosis. Altogether, these effects promoted LV dilatation in the longer term, as reflected by the increased scar circumference measured at 3 months (Fig. 3o).

### MF-specific AMPKα1 deficiency promotes CF activity

**Fig. 4:**
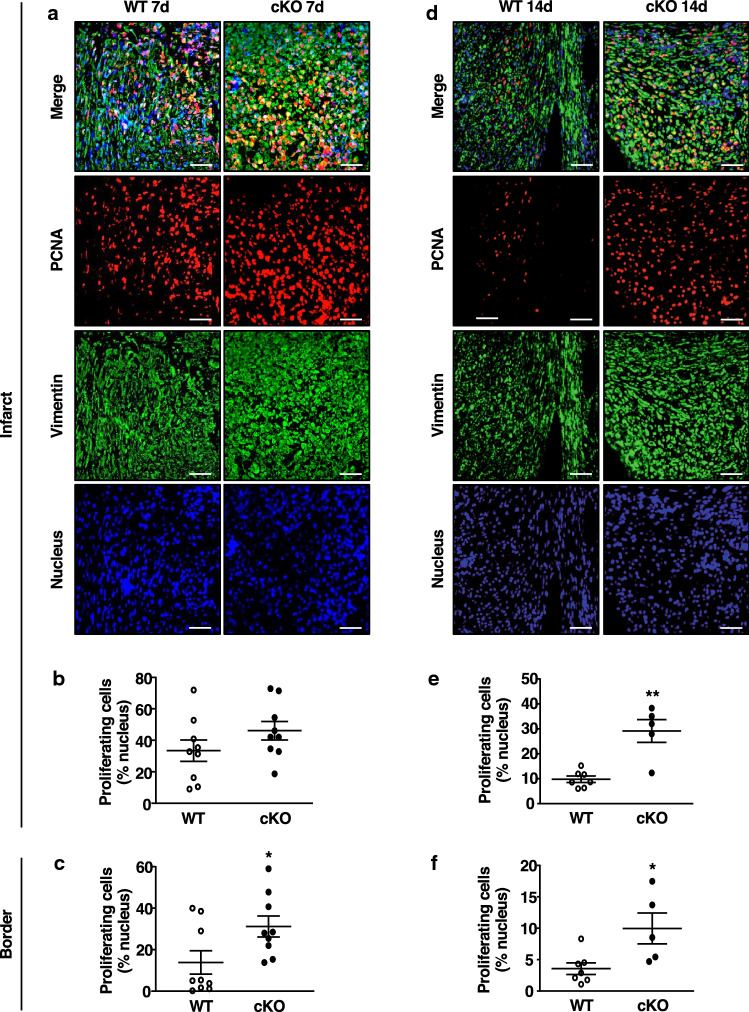
MF-specific AMPKα1 deficiency promotes CF activity. **a**, **d** Representative images of co-immunofluorescent staining of proliferating CFs (PCNA-positive: red fluorescence, vimentin-positive: green fluorescence) in the infarct area of MF-specific AMPKα1 KO (cKO) and littermates WT (WT) at 7 (**a**) and 14 (**d**) days post-myocardial infarction (MI). **b**, **c** Quantification of proliferating CFs in the infarct (**b**) or border (**c**) areas of cKO and WT at 7 days post-MI (*n* = 9 mice/group). **e**, **f** Quantification of proliferating CFs in the infarct (**e**) or border (**f**) areas of cKO and WT at 14 days post-MI (*n* = 5–7 mice/group). Results are expressed as % of PCNA-positive nucleus within the vimentin-stained tissue area. Magnification: ×20. Scale bars: 50 µm. Dot-plots represent data from individual mice, as well as mean ± SEM. Statistical significance was determined by unpaired *t* test (**b**, **c**, **f**) or Mann–Whitney test (**e**). *p < 0.05, **p < 0.01 compared to WT

MF AMPKα1 deletion substantially stimulated the fibrotic response, but underlying cellular mechanisms remain largely unknown. We thus examined CF proliferation using fluorescent immunohistochemistry of proliferating cell nuclear antigen (PCNA) proliferation marker, and vimentin which is commonly used to label interstitial cells and allows to demarcate the infarcted and border areas (Fig. 4a–f). At 7 days post-MI, immunohistochemistry revealed a non-significant trend towards increased proliferation in cKO infarcts, as compared to WT hearts (Fig. 4a, b), while we observed a number of PCNA-positive cells significantly higher in the border zone of cKO (Fig. 4c). Compared to 7 days post-MI, the number of proliferative cells at 14 days after MI was decreased (Fig. 4d–f) but remained significantly higher in both the infarct (Fig. 4e) and border (Fig. 4f) zones of cKO, as compared to WT hearts. The MF staining for αSMA (Suppl Fig. 5a–c) confirmed that, 2 weeks after MI, MFs were significantly more abundant in cKO infarct (Suppl Fig. 5b) and border zones (Suppl Fig. 5c). While it is recognised that fibroblast proliferation declines from days 5 to 7 post-MI to give way to the maturation phase, our results suggest that loss of AMPKα1 gene function in MFs promotes and sustains their proliferation in cKO infarcts, increasing their number, which underlies the enhanced fibrosis in mice with Postn-mediated MF-specific deletion.

### AMPKα1 regulates Cx43 expression in CFs

**Fig. 5:**
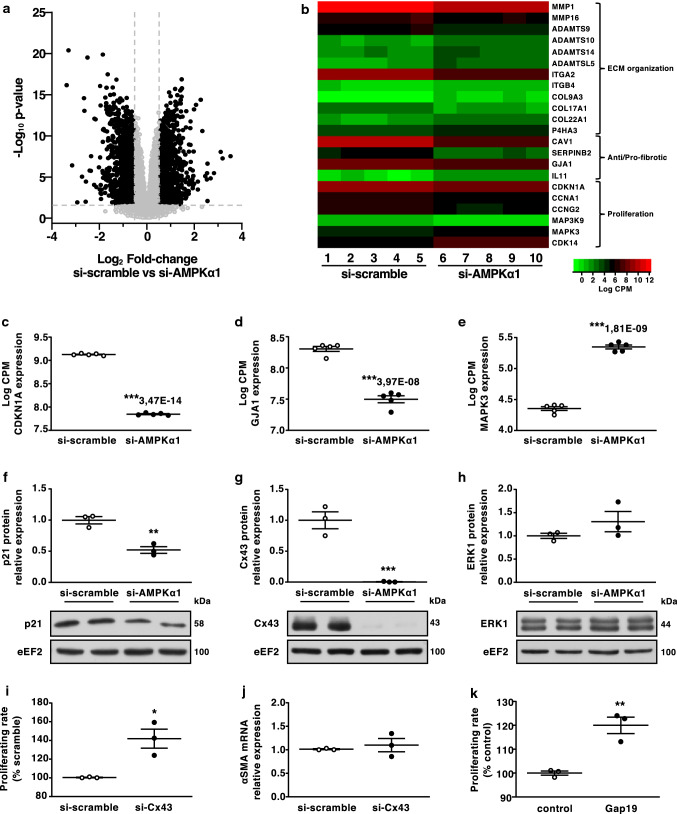
AMPKα1 regulates Cx43 expression in CFs. **a**–**h** Human cardiac fibroblasts (HCFs) were transfected with AMPKα1-targeting siRNA or scramble for 48 h and differential genes expression was analysed by RNA-Seq (*n* = 5 biological replicates/group) (**a**–**e**) or protein expression was assessed by Western blotting (*n* = 3 biological replicates/group) (**f**–**h**). **a** Volcano plot representation of differentially expressed genes. **b** Heat map representation of differentially expressed genes relevant for fibrosis and CF activity modulation. **c**–**h** Expression of mRNA (**c**–**e**) and corresponding proteins (**f**–**h**) relevant for CFs proliferation modulation. **i**–**j** HCFs were transfected with Cx43-targeting siRNA or scramble for 48 h and proliferation was assessed by flow cytometry measurement of EdU incorporation (*n* = 3 biological replicates/group) (**i**) or αSMA expression wad assessed by RT-qPCR (*n* = 3 biological replicates/group) (**j**). **k** HCFs were treated with Gap19 100 µM for 48 h and proliferation was assessed by flow cytometry measurement of EdU incorporation (*n* = 3 biological replicates/group). Dot plots represent data from biological replicates as well as mean ± SEM. Statistical significance was determined using false discovery rate (FDR) calculation (**a**–**e**), unpaired *t* test (**f**, **h**, **i**, **k**) or Mann–Whitney test (**g**, **j**). *p < 0.05, **p < 0.01, ***p < 0.001 compared to corresponding si-scramble or control

To identify AMPKα1-dependent gene regulation in fibroblasts, we performed a RNA-sequencing analysis in human CFs (HCFs) transfected with specific small interfering-RNA (siRNA) targeting AMPKα1 or control non-targeting siRNA (si-scramble). A total of 7375 genes, representing 53% of the analysed coding transcriptome, showed a differential regulation between both conditions, supporting the notion that AMPKα1 dramatically influences CF biology (GEO accession number: GSE147470). We selected the 1,846 genes presenting a fold change ≥ 1.5 and a statistical *p* value < 0.01 (Fig. 5a). Among these, we identified several dysregulated genes relevant for fibrosis and/or CF activity regulation including fibrotic factors, collagens and proteases which are demonstrated in the heat map view (Fig. 5b). We also found genes related to cellular proliferation and subsequently focused on CDKN1A (p21), MAPK3 (Erk1) and GJA1 (Cx43) (Fig. 5c–e) for their described interaction with the AMPKα1 signalling [1, 7, 8, 13, 40, 72]. We thus confirmed the RNA-sequencing output for these 3 genes at protein level. Western blot data confirmed that AMPKα1 deficiency decreased p21 and even more drastically Cx43 protein expression (Fig. 5f, g). In contrast, Erk1 expression was not different between both conditions (Fig. 5h). Several studies have linked reduced Cx43 expression with excessive fibrosis due to increased fibroblastic activity [29, 75, 76]. Accordingly, HCFs transfected with a specific siRNA targeting Cx43 proliferated faster than fibroblasts transfected with si-scramble (Fig. 5i), while expression of αSMA was not affected in these conditions (Fig. 5j). To further assess the contribution of Cx43 in the regulation of cardiac fibroblast proliferation, we used the mimetic peptide Gap19, a hemichannel blocker derived from the second cytoplasmic loop of Cx43 [28]. Results show that the HCF proliferation rate is significantly increased by the peptide treatment (Fig. 5k), supporting a central role of Cx43 hemichannels in the regulation of this process.

### AMPKα1 regulates Cx43 transcription and Cx43 mRNA degradation by a mechanism involving miR-125b-5p

**Fig. 6:**
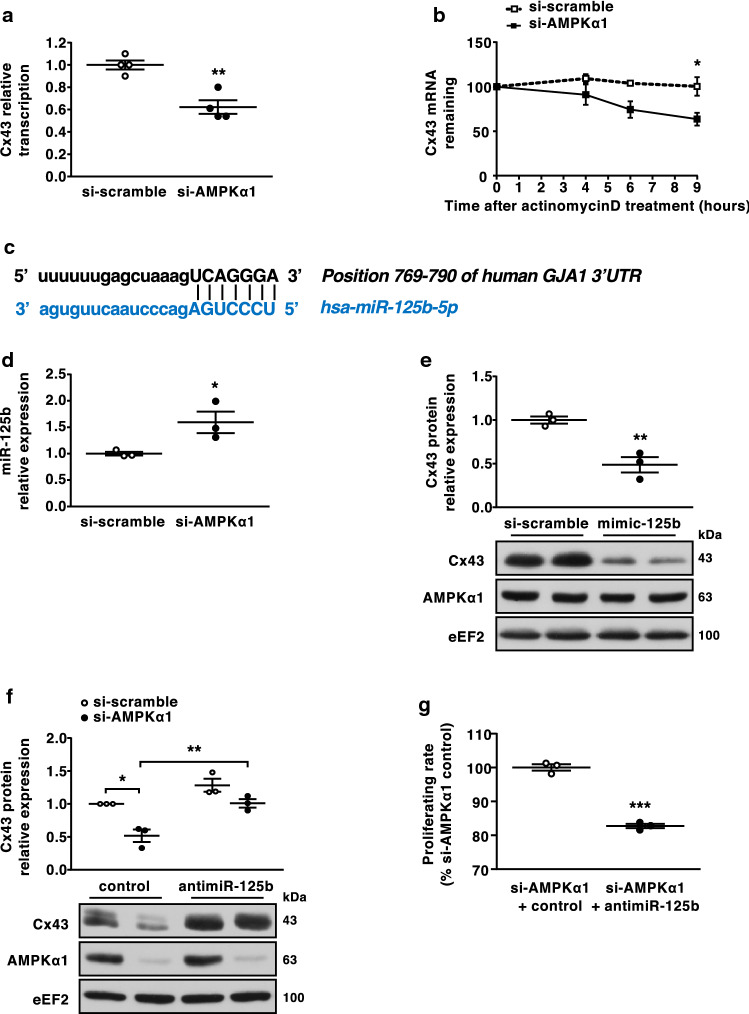
AMPKα1 regulates Cx43 transcription and Cx43 mRNA degradation by a mechanism involving miR-125b-5p.** a** Cx43 promotor activity measurement in AMPKα1-deficient or control HEK293 cells. HEK293 cells were co-transfected with a luciferase-reporter plasmid and AMPKα1-targeting siRNA or scramble (*n* = 4 biological replicates/group). Results are expressed as luciferase activity relative to scramble. **b** Human cardiac fibroblasts (HCFs) were transfected with AMPKα1-targeting siRNA or scramble for 48 h before being treated with actinomycin D 5 µg/mL. Cx43 expression was analysed at the different time points by RT-qPCR (*n* = 3 biological replicates/group). Results are expressed as the mean of fold change over corresponding basal state. **c** In silico determination of miR-125b-5p predicted target sites in the 3′UTR of Cx43 mRNA by Diana tools, Pictar and microRNA.org. **d** HCFs were transfected with AMPKα1-targeting siRNA or scramble for 48 h and miR-125b-5p expression was assessed by RT-qPCR (*n* = 3 biological replicates/group). Results are expressed as fold change over scramble. **e** HCFs were transfected with miR-125b-5p mimic or control for 48 h, with Cx43 expression analysed by Western blotting (*n* = 3 biological replicates/group). **f** HCFs were transfected with AMPKα1-targeting siRNA or scramble along with antagomir-125b-5p or control and Cx43 expression was evaluated by Western blotting (*n* = 3 biological replicates/group). **g** HCFs were transfected with AMPKα1-targeting siRNA or scramble along with antagomir-125b-5p or control for 48 h and proliferation was assessed by flow cytometry measurement of EdU incorporation (*n* = 3 biological replicates/group). Dot-plots represent data from biological replicates as well as mean ± SEM. Significance was determined by unpaired *t* test (**a**, **d**, **e**, **g**) or two-way ANOVA followed by Sidak’s multiple comparisons test (**b**, **f**). *p < 0.05, **p < 0.01, ***p < 0.01 compared to corresponding si-scramble or control

We sought to determine how AMPKα1 could interfere with Cx43 expression. To determine whether reduced expression was regulated at the transcriptional level, HEK293 cells were co-transfected with a Cx43 promotor-luciferase-reporter plasmid and AMPKα1-targeting or scrambled control siRNAs. Similar to HCFs, AMPKα1-deficient HEK293 exhibited decreased Cx43 protein and mRNA levels (Suppl. Figure 6a and 6b). As shown in Fig. 6a, AMPKα1 deletion decreased Cx43 promotor activity by ~ 40% compared to si-scramble, indicating that AMPKα1 regulates Cx43 transcription. However, this transcriptional regulation alone did not account for the nearly complete loss of Cx43 protein expression upon AMPKα1 deletion (Fig. 5g), suggesting that post-transcriptional mechanisms may also be involved.

We then explored whether the loss of AMPKα1 gene function affected Cx43 protein or mRNA stability. We observed no difference between AMPKα1 siRNA and scramble-transfected HCFs concerning Cx43 protein degradation by the proteasome (Suppl. Figure 7). To examine Cx43 mRNA stability, we incubated HCFs with actinomycin D for 9 h to inhibit RNA polymerase II and de novo transcription, and performed RT-qPCR at different time points to measure Cx43 mRNA levels. We observed a significant reduction in Cx43 mRNA stability in AMPKα1 siRNA-transfected cells compared with scrambled control siRNAs (Fig. 6b). We hypothesised that Cx43 mRNA downregulation in AMPKα1-deficient cells might be caused by an inhibitory microRNA that targets Cx43 mRNA. To gain an overview of changes in microRNA expression, a preliminary quantitative PCR microRNA array, containing 83 of the microRNAs common to most cardiovascular diseases, was performed in AMPKα1 siRNA-transfected HCFs. We found 43 microRNAs potentially up-regulated in AMPKα1-depleted HCFs (Suppl. Table 1). In silico analysis using the Diana, microRNA.org, and Pictar algorithms identified miR-125b-5p as having the potential to bind 7 bases of the Cx43 3′UTR mRNA at a site that is highly conserved across 11 species (Fig. 6c). Using RT-qPCR, we confirmed increased expression of miR-125b-5p in AMPKα1-deficient HCFs (Fig. 6d). To investigate whether miR-125b-5p downregulates Cx43 protein levels, HCFs were transfected with a miR-125b-5p mimic or scramble control for 48 h. Cx43 protein levels were reduced by 50%, as compared to control levels in cells transfected with the miR-125b-5p mimic (Fig. 6e). Very interestingly, reducing miR-125b-5p levels with miR-125b-5p antagomir counteracted the profound decrease in Cx43 protein expression as well as the stimulating effect on proliferation induced by AMPKα1 deletion in AMPKα1-deficient cells (Fig. 6f, g). Taken together, these data identify miR-125b-5p as an essential negative regulator of Cx43, contributing to the significantly reduced Cx43 expression in AMPKα1-deficient cells.

### AMPKα1 regulates Cx43 expression in infarcted areas and isolated fibroblasts of MF-specific AMPKα1 KO mice

**Fig. 7:**
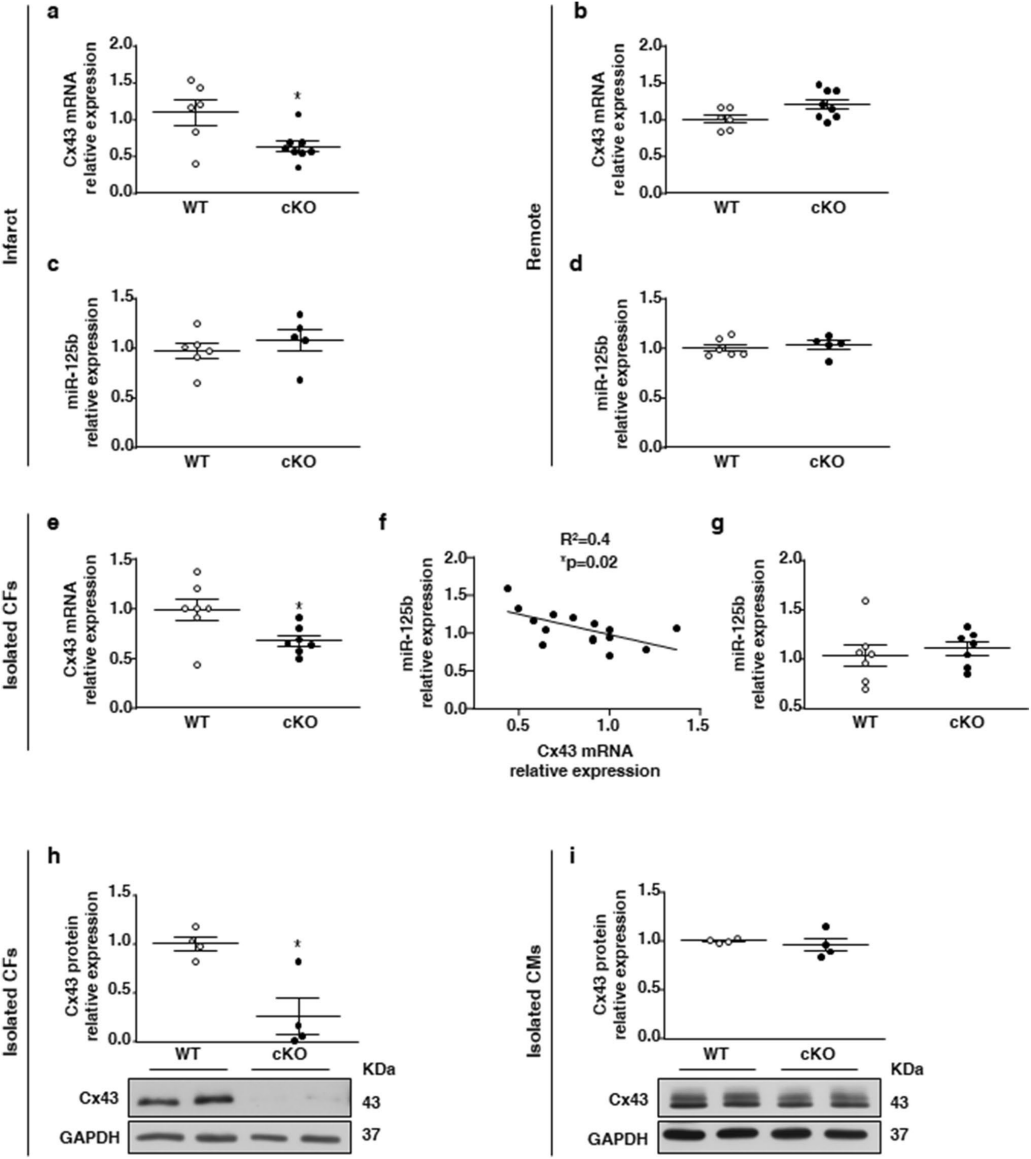
AMPKα1 regulates Cx43 expression in infarcted areas and isolated fibroblasts of MF-specific AMPKα1 KO mice.** a**–**d** qRT-PCR measurement of Cx43 mRNA (**a**, **b**) and miR-125b-5p (**c**, **d**) expression in the infarcted (**a**, **c**) and remote (**b**, **d**) myocardium of MF-specific AMPKα1 KO (cKO) and littermates WT (WT) mice at 7 days post-myocardial infarction (MI) (*n* = 5–8 mice/group). **e**,** g** qRT-PCR measurement of Cx43 mRNA (**e**) and miR-125b-5p (**g**) expression in isolated MFs from cKO and WT hearts at 3 days post-MI (*n* = 7 mice/group). **f** Correlation between Cx43 and miR-125b expressions in MFs isolated from cKO and WT hearts at 3 days post-MI (*n* = 14). **h**,** i** Western blot analysis of Cx43 protein expression in isolated MFs (**h**) or cardiomyocytes (CMs) (**i**) from cKO and WT hearts at 14 days post-MI (*n* = 4 mice/group). Dot plots represent data from individual mouse hearts, as well as mean ± SEM. Statistical significance was determined by unpaired *t* test (**a**–**e**, **g**, **h**) or Mann–Whitney test (**i**) and correlation was assessed using the Pearson coefficient *r* calculation (**f**). *p < 0.05 compared to WT

To determine in vivo the impact of the MF-specific AMPKα1 deletion on Cx43 and miR-125b-5p, their expression was first measured by qRT-PCR in total extracts from infarcted and remote areas of WT and cKO hearts. In the cKO infarcts, Cx43 mRNA expression was 50% lower at day 7 post-MI, as compared to infarcts of WT littermates (Fig. 7a). In contrast, no genotype-related differences were noted in the remote zone, suggesting that non-fibroblastic cells of either genotype express unaltered Cx43 levels (Fig. 7b). Expression level of miR-125b-5p in the infarcted or remote areas was not affected at this time point (Fig. 7c, d). We then evaluated Cx43 and miR-125b-5p expression in MFs isolated from WT and cKO infarcted hearts, at 3 days post-MI. In agreement with the in vitro results, Cx43 mRNA expression was robustly reduced in MFs isolated from cKO infarcted hearts (Fig. 7e) and was inversely correlated with the level of miR-125b-5p expression (Fig. 7f). However, miR-125b-5p expression was not significantly different in MFs isolated from cKO and WT hearts (Fig. 7g), suggesting a likely fine-tuning relationship between miR-125b-5p and Cx43. Finally, Cx43 protein expression was measured at 14 days post-MI (Fig. 7h, i), and we confirmed that it was robustly reduced in cKO, as compared to WT MFs (Fig. 7h), while it remained comparable in WT and cKO isolated cardiomyocytes (Fig. 7i).

## Discussion

In this study, we highlight the crucial role of MF-AMPKα1 in controlling MF properties, scar formation, and LV remodelling post-MI. Mice with MF-specific deletion of AMPKα1 develop a more tightly-packed collagen scar after MI with a hyperproliferative fibroblast response, and suffer from adverse LV remodelling and functional impairment in the first months after MI. We provide evidence that Cx43 is downregulated in MFs isolated from the acutely injured myocardium, probably contributing to the pro-fibrotic responses. Mechanistically, we show that AMPKα1 mainly influences Cx43 expression by a transcriptional mechanism. We also identified a microRNA, miR-125b-5p, which can play a fine-tuning role to silence Cx43 and serve as a positive regulator in the fibrotic remodelling process [47].

Our previous observations had revealed that systemic AMPKα1 invalidation enhanced adverse LV remodelling by increasing fibroblast proliferation, while myodifferentiation and scar maturation were impaired [49]. We thus hypothesised that fibroblastic AMPKα1 was a key signalling element in regulating fibrosis in the infarcted myocardium and an attractive target for therapeutic intervention. Here, we employed a mouse model in which Cre-recombinase is driven by a mouse Postn promoter for selective AMPKα1 deletion in MFs. Indeed, Postn is a recognised MF marker, and is activated in CF in response to numerous cardiac stimuli invoking a fibrotic response, including necrosis [19, 31]. Three studies have previously investigated the role of MFs in MI-induced fibrotic remodelling using the Postn-Cre model. Specific MF-invalidation of glycogen synthase kinase 3β (GSK-3β) was associated with hyperactivation of canonical TGF-β signalling, resulting in excessive fibrosis and LV dilatation in a mouse MI model [37]. In contrast, MF-specific downregulation of Sox9 reduced ECM deposition in infarcted hearts [60]. Finally, Kong et al. recently reported that cell-specific activation of Smad3 signalling in cardiomyocytes and CFs differentially regulates repair and remodelling of the infarcted heart [35]. Using R26-stop^fl/fl^-EYFP reporter animals, we clearly demonstrated that Cre-recombinase is selectively expressed in interstitial cells of infarcted areas, validating the use of Postn-Cre model. AMPKα1-specific deletion in MFs is associated with stimulating proliferation post-MI. MF hyperactivity leads to excessive fibrosis that aggravates MI-induced LV dilatation. cKO mice subjected to MI are characterised by increased collagen deposition and density in infarct core and border zones, without changes in cardiac LOX expression or fibres cross-linking. These observations contrast with the AMPKα1 systemic KO mouse phenotype, with its markedly reduced myocardial LOX expression, resulting in defective collagen scar maturation [49]. The genotype-related difference could be accounted for by non-CF cardiac cells in our current study, which still express AMPKα1, maintain LOX expression, and thus contribute to collagen maturation, even in the absence of AMPKα1 in MFs. Though AMPKα1 systemic KO mice exhibit reduced LOX expression and collagen maturation, MF-specific deletion of AMPKα1 results in enhanced fibrosis and well-aligned and thickly packed collagen fibres, with exacerbated LV dilatation and impaired cardiac function. An over-compliant scar has been well recognised to cause LV dilatation and even cardiac rupture [49, 68, 69]. Inversely, excessive collagen type I deposition or maturation has been reported to increase LV wall stress, along with exacerbated compensation and LV dilatation [21, 37, 66]. It is, therefore, not surprising that the two genotypes studied (systemic KO and cKO) exhibited a deleterious phenotype and exacerbated LV remodelling, albeit via different fibrotic responses.

Interestingly, increased fibrosis in cKO hearts is associated with markedly decreased Cx43 expression in MFs. Several studies conducted in non-cardiac tissues have associated a reduction in Cx43 expression with increased collagen deposition [9, 45, 54]. Regarding the heart, Cx43 is highly expressed in cardiomyocytes and CFs [44]. Abnormal cardiac Cx43 expression has been reported in human ischaemic and dilated cardiomyopathies [16]. Mitochondrial Cx43 was even demonstrated to play a role in cardiomyocyte ischaemic conditioning by modulating K^+^ influx and ROS production [22]. In addition, partial Cx43 deficiency has been associated with an exaggerated fibrotic response in mice submitted to pressure overload through transverse aortic constriction, or to chronic Angiotensin II treatment [29, 67]. Finally, this inverse relationship between Cx43 expression and cardiac fibrosis has been recently demonstrated in rats submitted to MI and treated with Dexmedetomidine, an α2-adrenoceptor agonist [71]. Mechanistically, Cx43 expression levels were shown to be related to CF proliferation [29, 75, 76]. Interestingly, tumour cells exhibit similar effects. Various studies have described that diminished levels of Cx43 were inversely correlated to tumour proliferation [2, 27, 57, 62]. In line with our findings, Cx43 expression was shown to be downregulated by miR-125b-5p through its binding with Cx43 mRNA 3′ UTR in glioma cells [3, 30]. Together, these studies support an important role for Cx43 in fibroblast activation and proliferation. At present, the molecular mechanism underlying the increased proliferation remains unknown. Our data with the mimetic peptide Gap19 support a role of Cx43 hemichannels in the regulation of this process. By facilitating bidirectional passage over the plasma membrane of ions and signalling or metabolic molecules, Cx43 hemichannels may modulate various biological responses [32]. Of note, previous studies have shown that Cxs fragments can be found within the nucleus and control transcription of genes regulating cell proliferation [10, 36]. Regarding the link between AMPK and Cx43, Li et al*.* reported that AMPK activation likely protects cardiac gap junctions from remodelling under hyperglycemia [40]. Our data strongly argue in favour of AMPK and Cx43 intervening in the fibrotic process. Our hypothesis is that AMPKα1 has not to be overexpressed or activated to regulate MF proliferation and restrain fibrosis, but can exert its function in a basal state, through non-catalytic mechanisms such as scaffolding of protein complexes, protein interactions or subcellular targeting [55]. These non-catalytic functions might explain how AMPKα1 influences Cx43 expression in MFs, and consequently MF activity, by both a transcriptional and a post-transcriptional mechanism involving miR-125b-5p. The latter was previously shown to be up-regulated in mouse and human fibrotic hearts, while playing a functional role in cardiac fibrogenesis [47]. Several studies have reported on AMPK-dependent microRNA regulation. Recently, the transcriptional regulation of miR-184 by AMPK has been demonstrated in pancreatic islets [42]. In addition, AMPK has proven to indirectly regulate microRNAs by phosphorylating p53 or increasing expression of β-arrestin, two proteins involved in their post-transcriptional processing [34, 39, 70]. Although our in vitro results clearly show an AMPK-dependent regulation of miR-125b-5p in fibroblasts, future work will aim to confirm that this regulation occurs in vivo and to determine the precise mechanism by which AMPKα1 affects the levels of this microRNA.

Although our study focused on the AMPKα1-Cx43 axis, RNA-sequencing analysis carried out on AMPKα1-deficient HCFs highlighted AMPKα1 as a key signalling element regulating a plethora of fibrotic genes. For example, MMP-1, IL-11, or Caveolin-1 are strongly modulated in AMPKα1-deficient HCFs and considered as key fibrotic factors [43, 59]. Therefore, their possible contribution to the pro-fibrotic phenotype observed in MF-specific AMPKα1 KO mice cannot be excluded.

A limitation of our study is the use of LAD coronary artery permanent ligation model to produce a large scar and severe remodelling, while the ischaemia/reperfusion (I/R) model reflects better the clinical situation observed in human following an acute MI. I/R can confer other advantages and it will be interesting to investigate whether fibroblastic AMPKα1 can also play important roles in this condition where the temporal dynamics of the inflammatory response and the ROS-related oxidative stress might be playing a greater role during the development of cardiac remodelling and heart failure.

In conclusion, our work adds momentum to the emerging notion that dampening MF in a timely fashion, that does not interfere with its role in cardiac repair, may be a new therapeutic strategy in heart failure prevention. Here, we highlight a novel central regulator of CF activity—AMPKα1—that might constitute a potential target for pharmacological anti-fibrotic applications.

## Supplementary Information

Below is the link to the electronic supplementary material.

Supplementary file1 (PDF 65 KB)

Supplementary file2 (PDF 32 KB)

Supplementary file3 (PDF 11077 KB)

## Data Availability

Raw and processed RNA-seq data were deposited and made publicly available on the Gene Expression Omnibus (GSE147470).
