# Supplementary material for: AMPKα1 deletion in myofibroblasts exacerbates post-myocardial infarction fibrosis by a connexin 43 mechanism
Source: Basic Res Cardiol. 2021 Feb 9;116(1):10. doi: 10.1007/s00395-021-00846-y (PMC7873123; doi:10.1007/s00395-021-00846-y)
Supplement: Supplementary file 1 — Supplementary file1 (PDF 65 KB) [file 395_2021_846_MOESM1_ESM.pdf]

Full Title

AMPKα1 deletion in myofibroblasts exacerbates post myocardial infarction fibrosis by a Connexin 43 mechanism

Authors and affiliations

Cécile Dufeys[1,11], Evangelos-Panagiotis Daskalopoulos[1,11], Diego Castanares-Zapatero[1], Simon J. Conway[2], Audrey Ginion[1], Caroline Bouzin[3], Jérôme Ambroise[4], Bertrand Bearzatto[4], Jean-Luc Gala[4], Stephane Heymans[5], Anna-Pia Papageorgiou[5,6], Stefan Vinckier[7], Julien Cumps[1], Jean-Luc Balligand[8], Maarten Vanhaverbeke[6,9], Peter Sinnaeve[6,9], Stefan Janssens[6,9], Luc Bertrand[1], Christophe Beauloye[1,10], and Sandrine Horman[1,*]

[1]Pôle de Recherche Cardiovasculaire (CARD), Institut de Recherche Expérimentale et Clinique (IREC), Université catholique de Louvain (UCLouvain), Brussels, Belgium

[2]HB Wells Center for Pediatric Research, Indiana University School of Medicine, Indianapolis, IN, USA

[3]IREC Imaging Platform, Institut de Recherche Expérimentale et Clinique (IREC), Université catholique de Louvain (UCLouvain), Brussels, Belgium

[4]Centre de Technologies Moléculaires Appliquées, Institut de Recherche Expérimentale et Clinique, UCL, Brussels, Belgium

[5]Center for Heart Failure Research, Cardiovascular Research Institute Maastricht (CARIM), Maastricht University, Maastricht, The Netherlands

[6]Department of Cardiovascular Sciences, KU Leuven, Leuven, Belgium

[7]Center for Cancer Biology, University of Leuven and VIB, Leuven, Belgium

[8]Pôle de Pharmacologie et de Thérapeutique (FATH), Institut de Recherche Expérimentale et Clinique (IREC), Université catholique de Louvain (UCLouvain), Brussels, Belgium

[9]Department of Cardiovascular Medicine, Leuven University Hospitals, Leuven, Belgium

[10]Cliniques Universitaires Saint-Luc, Division of Cardiology, Brussels, Belgium

[11]These authors contributed equally

Corresponding author

Prof. Sandrine Horman, PhD

Pôle de Recherche Cardiovasculaire (CARD)

Institut de Recherche Expérimentale et Clinique (IREC)

Université catholique de Louvain (UCLouvain)

55, Avenue Hippocrate

B-1200, Brussels, Belgium

sandrine.horman@uclouvain.be

+32 2 764 55 66

Supplemental Material and Methods

Reagents

Tribromoethanol (#T48402), phosphomolybdic acid (#79560), osmium tetroxide (#75632), liberase DH research grade (#LIBDGRO), laminin (#L2020), actinomycin D (#A9415) and anti-lysyl oxidase antibody (#L4669) were purchased from Sigma-Aldrich. Entellan (#108690500) and anti-AMPKα1 antibody (#07350) were obtained from Merck Millipore. Temgesic was from Indivior. Dolethal came from Vetoquinol. Avidin/biotin blocking kit (#SP2001), biotinylated griffonia simplicifolia lectin I isolectin B4 (#B1205), fluorescein avidin D (#A2001), rhodamine labeled wheat germ agglutinin (WGA) (#RL1022) and vectashield mounting medium with DAPI (#H1200) were purchased from Vector Laboratories. Bovine serum albumin (BSA) (#8076) was from Roth. RNA 6000 nano kit (#5067-1511), EnVision system-HRP labelled polymer anti-rabbit (#K4003) and Dako fluorescence mounting medium (#S3023) were obtained from Agilent. Type II collagenase (#LS004177) was from Worthington. L-glutamine (#25030081), trypsin (#15090046), penicillin/streptomycin (#15070063) and Opti-MEM medium (#31985047) were sourced from Gibco. Human cardiac fibroblasts (HCFs) were from ScienCell Reserch Laboratories (#6300).) HCF basal medium (#315500) and HCF growth supplement (#316GS) were purchased from Cell applications. Small interfering RNA (siRNA) targeting PRKAA1 (#AM51334), siRNA targeting GJA1 (#AM16708), siRNA negative control (#AM4635), has-miR-125b-5p miRvana miRNA inhibitor (#4464084) and miRvana miRNA inhibitor negative control (#4464074) were obtained from Ambion. Alexa fluor-488 (#B40953) or -555 (#B40955) tyramide reagent, Click-iT Edu alexa fluor 488 flow cytometry assay kit (#C10420), lipofectamine RNAi max (#13778150), Hoechst 33342 (#62249), halt protease and phosphatase inhibitor cocktail (#78446), foetal bovine serum (FBS) (#SV3016003), Qubit RNA BR assay kit (#10211) and anti-AMPKα2 antibody (#PA521494) were sourced from Thermo Fischer Scientific. pLightSwitch-GJA1 (#S700799) or -empty control (#S790005) promotor reporter GoClone plasmid, LightSwitch luciferase assay kit (#LS010) and DharmaFECT duo transfection reagent (#T201001) were from SwitchGear Genomic. Syn-hsa-miR-125b-5p miScript miRNA mimic (#MSY0000423), RNeasy mini kit (#74104), miRNeasy mini kit (#217004), DNase (#79254), miScript II RT kit (#218161), miScript SYBR green PCR kit (#218073) and miScript miRNA PCR Array Human cardiovascular disease (#331221) were

purchased from Qiagen. iScript cDNA synthesis kit (#1708891) came from Bio-Rad. qPCR core kit for SYBR green I (#RTSN1005NR) was sourced from Eurogentec. MG-132 (#S2619) was from Selleckchem. Anti-Cx43 (#3512), anti-GAPDH (#5174), anti-PCNA (#13110), anti-p21 (#2947), anti-ERK1/2 (#9102) and anti-eEF2 (#2332) antibodies were purchased from Cell Signaling.. Anti-αSMA (#ab124964) and anti-vimentin (#ab92547) antibodies were obtained from Abcam. Anti-GFP antibody (#NB600308) was from Novus Biologicals. KAPA RNA HyperPrep Kit with RiboErase (#KK8560) came from KAPA Biosystems. Gap 19 (#5353) was from Tocris.

Immunostaining for αSMA in isolated mouse cardiac fibroblasts

Isolated mouse CFs were fixed with 4% paraformaldehyde for 5 minutes. After washing with PBS, cells were permeabilized in PBS / Triton-X 0.3% for 15 minutes and blocked for 30 minutes with PBS / Triton-X 0.3% / 5% BSA. Anti-αSMA antibody (1:300) was incubated overnight at 4°C. After washing in PBS, secondary antibody was incubated for 45 minutes before washing and mounting in vectashield with DAPI.

Tissue processing before histological analysis

The hearts were excised, stopped at diastole with 250 mM KCl and rinsed with PBS. Hearts were then fixed with 4% paraformaldehyde for 24 hours at room temperature. Fixed tissues were paraffin embedded with the Tissue-Tek VIP6 (Sakura) and 5 µm-thick serial sections were made. Before each staining or immunochemistry, slices were deparaffinized and rehydrated.

Cardiomyocyte hypertrophy and capillary density assessment

After antigen retrieval by microwave heating for 15 minutes in citrate buffer pH 6, endogenous biotins were inhibited using avidin/biotin blocking kit according to the manufacturer's recommendations. Slides were incubated overnight with biotinylated isolectin B4 (1:150) at 4°C. After washing with PBS, slides were treated 2 hours with avidin-fluorescein (1:50) at room

temperature and washed with PBS. Heart sections were then incubated for 1 hour with WGA-rhodamin (1:50) before washing and mounting in vectashield with DAPI. Pictures were acquired with a Axio Imager microscope (Carl Zeiss) with a 40X objective. Cell size (>500 cells per sample) and vessel number per myocyte were determined using Axiovision software (Carl Zeiss).

Isolation of adult murine cardiomyocytes from cardiac muscle tissue

Hearts were perfused with 0.625mg liberase and 2.1mg trypsin as previously described [1]. Cardiomyocytes were then purified, cultured and plated in laminin-coated dishes in minimum essential medium with Hank's salts supplemented with 2mM L-glutamine , 10% BSA and 2% penicillin / streptomycin. After 2 hours, isolated cardiomyocytes were lysed for western blotting analysis.

Cx43 promotor activity assay

HEK293 cells were co-transfected with 100 ng of plasmid pLightSwitch-GJA1 or pLightSwitch-empty control reporter vector and 30 nM of AMPKα1 targeting siRNA or scramble using DharmaFECT Duo transfection reagent following the manufacturer's instructions. Luciferase activity was assayed after 24 hours using LightSwitch Luciferase Assay Reagent following the manufacturer's recommendations.

Proteasome analysis

Transfected HCFs were serum starved for 2 hours, treated with 20 µM MG-132 for 6 hours and lysed for western blotting analysis.

mRNA stability measurement

Transfected HCFs were serum starved for 2 hours, treated with 5 µg/ml actinomycin D for 0, 4, 6 and 9 hours and lysed for qRT-PCR analysis.

microRNAs array

Total microRNAs were extract and reverse transcribed as described in main methods. MicroRNAs PCR array for Cardiovascular Disease was performed and analyzed according to the manufacturer's instructions.

Western blotting

Cells and tissues were lysed in cold buffer containing 50 mM Hepes, 1 mM dithiothreitol, 50 mM KCl, 1% Triton X-100 and a cocktail of proteases and phosphatases inhibitors. Lysates were centrifuged at 15,000g for 15 minutes and protein concentration in the supernatant was measured by the Bradford method. Equal amounts of proteins were separated by sodium dodecyl sulfate-polyacrylamide gel electrophoresis (SDS-PAGE) and subsequently transferred to polyvinylidene difluoride (PVDF) membrane. The membranes were blocked with TBS / 0.01% Tween 20 / 5% BSA and probed overnight at 4°C with antibodies targeting AMPKα1, AMPKα2, Cx43, LOX, p21, ERK1/2 and eEF2 (1:1,000) or GAPDH (1:50,000). After incubation with the appropriate secondary antibody (1:20,000) for 1 hour at room temperature, proteins were visualized with chemiluminescence HRP substrate. Band intensities were quantified by Image J software (version 1.6, National Institutes of Health, Bethesda, MD, USA). GAPDH (*in vivo*) and eEF2 (*in vitro*) were used as loading controls. Band intensities were normalized relative to those of loading controls on the same gel and results are reported as densitometric relative expression.

List of primers

	Forward primer (5’-3’)	Reverse primer (5’-3’)
Human Cx43	ATGAGCAGTCTGCCTTTCGT	TCTGCTTCAAGTGCATGTCC
Human αSMA	ACTGGGACGACATGGAAAAG	GCGTCCAGAGGCATAGAGAG
Mouse Cx43	GTGGCCTGCTGAGAACCTAC	GAGCGAGAGACACCAAGGAC
Mouse LOX	GGAGGACACGTCCTGTACT	CCAGGTAGCTGGGGTTTACA

Mouse RPL32	GCCCAAGATCGTCAAAAAGA	ATTGTGGACCAGGAACTTGC
Human RPL32	AGGCATTGACAACAGGGTTC	GTTGCACATCAGCAGCACTT
mir-125b-5p	UCCCUGAGACCCUAACUUGUGA	

Reference

1. Van Steenbergen A, Balteau M, Ginion A, Ferte L, Battault S, Ravenstein CM, Balligand JL, Daskalopoulos EP, Gilon P, Despa F, Despa S, Vanoverschelde JL, Horman S, Koepsell H, Berry G, Hue L, Bertrand L, Beauloye C (2017) Sodium-myoinositol cotransporter-1, SMIT1, mediates the production of reactive oxygen species induced by hyperglycemia in the heart. Scientific reports 7:41166 doi:10.1038/srep41166
