# Supplementary material for: AMPKα1 deletion in myofibroblasts exacerbates post-myocardial infarction fibrosis by a connexin 43 mechanism
Source: Basic Res Cardiol. 2021 Feb 9;116(1):10. doi: 10.1007/s00395-021-00846-y (PMC7873123; doi:10.1007/s00395-021-00846-y)
Supplement: Supplementary file 2 — Supplementary file2 (PDF 32 KB) [file 395_2021_846_MOESM2_ESM.pdf]

Supplementary Table 1, related to Fig. 6 Dysregulated microRNAs in AMPKα1-deficient HCFs.

microRNAs	Fold change	p-value	microRNAs	Fold change	p-value
hsa-let-7a-5p	+2,35	0,46	hsa-miR-199a-5p	+2,11	0,36
hsa-let-7b-5p	+2,06	0,42	hsa-miR-21-5p	+2,11	0,45
hsa-let-7c-5p	+1,76	0,65	hsa-miR-210-3p	+4,69	0,37
hsa-let-7d-5p	+1,70	0,64	hsa-miR-214-3p	+1,89	0,10
hsa-let-7e-5p	+1,99	0,54	hsa-miR-221-3p	+1,58	0,62
hsa-let-7f-5p	+1,81	0,74	hsa-miR-224-5p	+2,11	0,69
hsa-miR-103a-3p	+2,67	0,35	hsa-miR-23a-3p	+2,13	0,49
hsa-miR-10b-5p	+2,28	0,77	hsa-miR-23b-3p	+1,80	0,56
hsa-miR-124-3p	+3,69	0,37	hsa-miR-25-3p	+1,71	0,50
hsa-miR-125a-5p	+1,66	0,63	hsa-miR-26a-5p	+1,51	0,56
hsa-miR-125b-5p	+1,56	0,11	hsa-miR-26b-5p	+2,34	0,52
hsa-miR-126-3p	+1,57	0,72	hsa-miR-27b-3p	+1,80	0,51
hsa-miR-140-5p	+2,31	0,10	hsa-miR-29a-3p	+1,51	0,24
hsa-miR-143-3p	+2,11	0,51	hsa-miR-29b-3p	+2,05	0,83
hsa-miR-145-5p	+2,43	0,18	hsa-miR-29c-3p	+1,79	0,19
hsa-miR-155-5p	+1,59	0,52	hsa-miR-31-5p	+3,43	0,32
hsa-miR-15b-5p	+1,82	0,54	hsa-miR-320a	+1,87	0,53
hsa-miR-16-5p	+2,17	0,04	hsa-miR-494-3p	+1,57	0,35
hsa-miR-106a-5p	+1,89	0,12	hsa-miR-7-5p	+1,64	0,58
hsa-mir-17-5p	+1,89	0,12	hsa-miR-93-5p	+1,78	0,35
hsa-miR-181a-5p	+1,96	0,02	hsa-miR-98-5p	+2,12	0,58
hsa-miR-181b-5p	+1,86	0,56			

Human CFs (HCFs) were transfected with AMPKα1-targeting siRNA or scramble for 48 hours, total microRNAs were extracted and analyzed by pre-designed miScript miRNA PCR Array for Human Cardiovascular Disease (n=3 biological replicate/group).
