# Supplementary material for: AMPKα1 deletion in myofibroblasts exacerbates post-myocardial infarction fibrosis by a connexin 43 mechanism
Source: Basic Res Cardiol. 2021 Feb 9;116(1):10. doi: 10.1007/s00395-021-00846-y (PMC7873123; doi:10.1007/s00395-021-00846-y)
Supplement: Supplementary file 3 — Supplementary file3 (PDF 11077 KB) [file 395_2021_846_MOESM3_ESM.pdf]

Full Title

AMPKα1 deletion in myofibroblasts exacerbates post myocardial infarction fibrosis by a Connexin 43 mechanism

Authors and affiliations

Cécile Dufeys[1,11], Evangelos-Panagiotis Daskalopoulos[1,11], Diego Castanares-Zapatero[1], Simon J. Conway[2], Audrey Ginion[1], Caroline Bouzin[3], Jérôme Ambroise[4], Bertrand Bearzatto[4], Jean-Luc Gala[4], Stephane Heymans[5], Anna-Pia Papageorgiou[5,6], Stefan Vinckier[7], Julien Cumps[1], Jean-Luc Balligand[8], Maarten Vanhaverbeke[6,9], Peter Sinnaeve[6,9], Stefan Janssens[6,9], Luc Bertrand[1], Christophe Beauloye[1,10], and Sandrine Horman[1,*]

[1]Pôle de Recherche Cardiovasculaire (CARD), Institut de Recherche Expérimentale et Clinique (IREC), Université catholique de Louvain (UCLouvain), Brussels, Belgium

[2]HB Wells Center for Pediatric Research, Indiana University School of Medicine, Indianapolis, IN, USA

[3]IREC Imaging Platform, Institut de Recherche Expérimentale et Clinique (IREC), Université catholique de Louvain (UCLouvain), Brussels, Belgium

[4]Centre de Technologies Moléculaires Appliquées, Institut de Recherche Expérimentale et Clinique, UCL, Brussels, Belgium

[5]Center for Heart Failure Research, Cardiovascular Research Institute Maastricht (CARIM), Maastricht University, Maastricht, The Netherlands

[6]Department of Cardiovascular Sciences, KU Leuven, Leuven, Belgium

[7]Center for Cancer Biology, University of Leuven and VIB, Leuven, Belgium

[8]Pôle de Pharmacologie et de Thérapeutique (FATH), Institut de Recherche Expérimentale et Clinique (IREC), Université catholique de Louvain (UCLouvain), Brussels, Belgium [9]Department of Cardiovascular Medicine, Leuven University Hospitals, Leuven, Belgium [10]Cliniques Universitaires Saint-Luc, Division of Cardiology, Brussels, Belgium

[11]These authors contributed equally

Corresponding author

Prof. Sandrine Horman, PhD

Pôle de Recherche Cardiovasculaire (CARD)

Institut de Recherche Expérimentale et Clinique (IREC)

Université catholique de Louvain (UCLouvain)

55, Avenue Hippocrate

B-1200, Brussels, Belgium

sandrine.horman@uclouvain.be

+32 2 764 55 66

Supplementary Figures

Supplementary Fig. 1

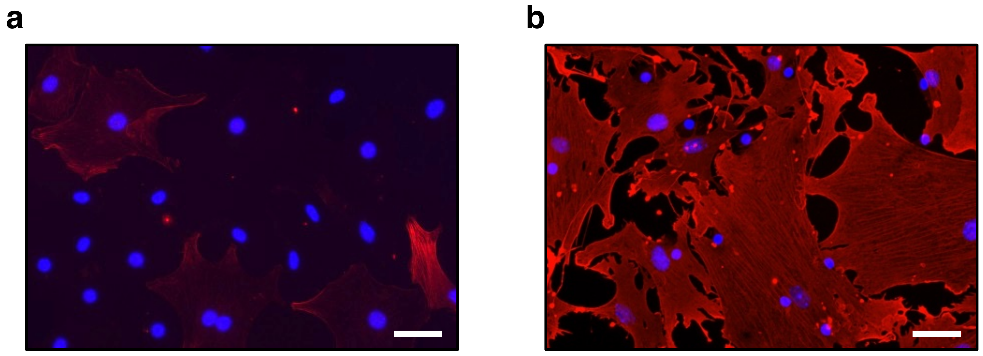

Supplementary Fig. 1, related to Fig. 1 Isolated cardiac interstitial non-myocyte cells are fibroblasts **a and b** Representative images of non-myocyte cells isolated from sham (a) or infarcted (b) mouse hearts. Fibroblasts were observed under fluorescent microscopy after immunostaining for αSMA. Magnification: 20x. Scale bars: 50µm.

Supplementary Fig. 2

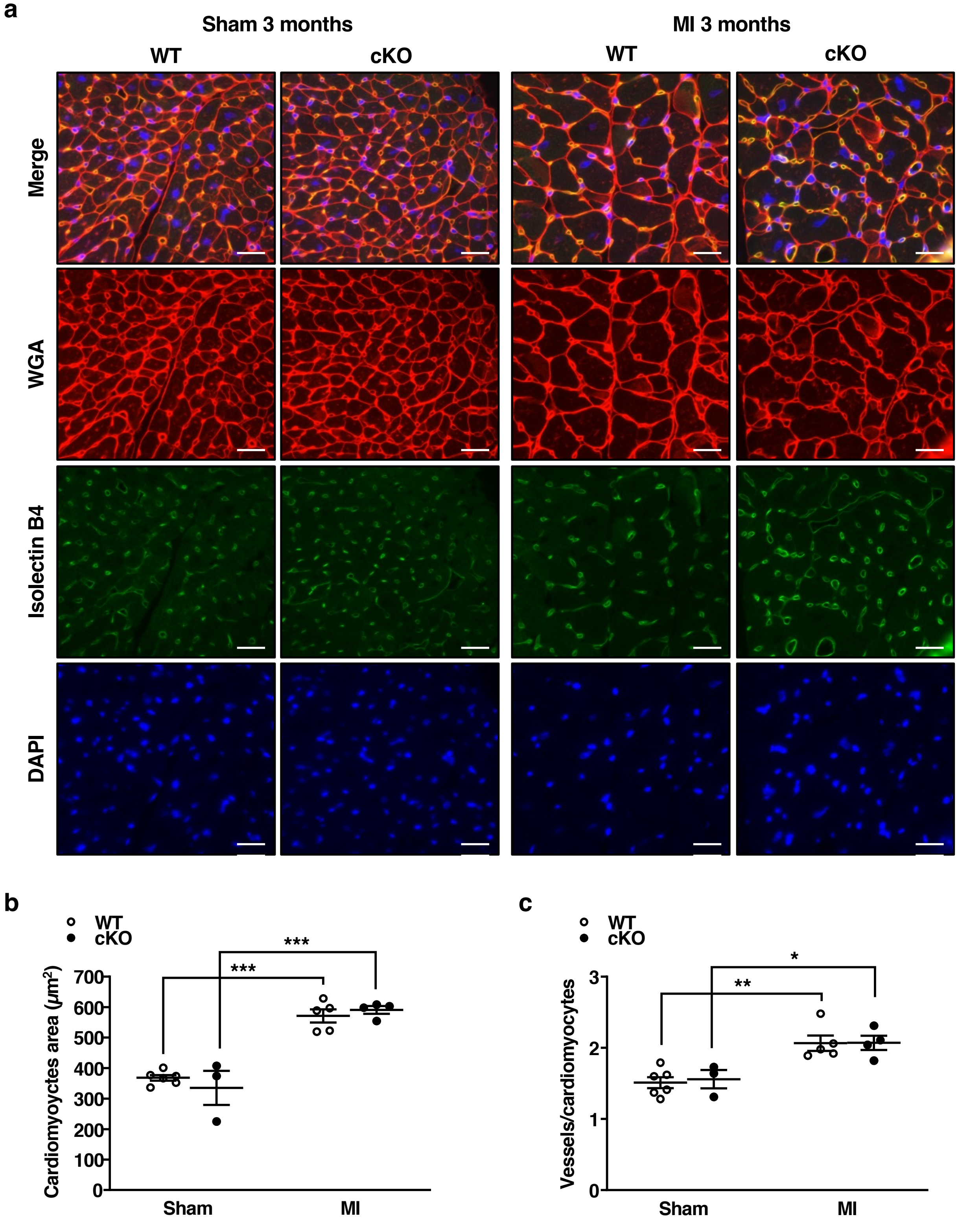

Supplementary Fig. 2, related to Fig. 2 MF-specific AMPKα1 deletion does not influence cardiomyocyte hypertrophy and angiogenesis in the remote area **a** Representative images of wheat-germ agglutinin (WGA) and isolectin B4 staining in MF-specific AMPKα1 KO (cKO) and littermate WT (WT) remote areas at 3 months after myocardial infarction (MI) or sham surgery. Magnification: 40x. Scale bars: 25µm. **b** Quantification of cardiomyocyte hypertrophy in the remote area of cKO and WT mice using WGA staining (n=3-6 mice/group). Results are expressed as mean of cardiomyocyte areas. **c** Quantification of vessel count in the remote area of cKO and WT mice using isolectin B4 staining (n=3-6 mice/group). Results are expressed as mean of vessels number per cardiomyocytes. (b and c) Dot-plots represent data from individual mice, as well as mean ± SEM. Statistical significance was determined using two-way ANOVA followed by Sidak's multiple comparisons test. *p<0.05, **p<0.01, ***p<0.001 compared to corresponding sham.

Supplementary Fig. 3

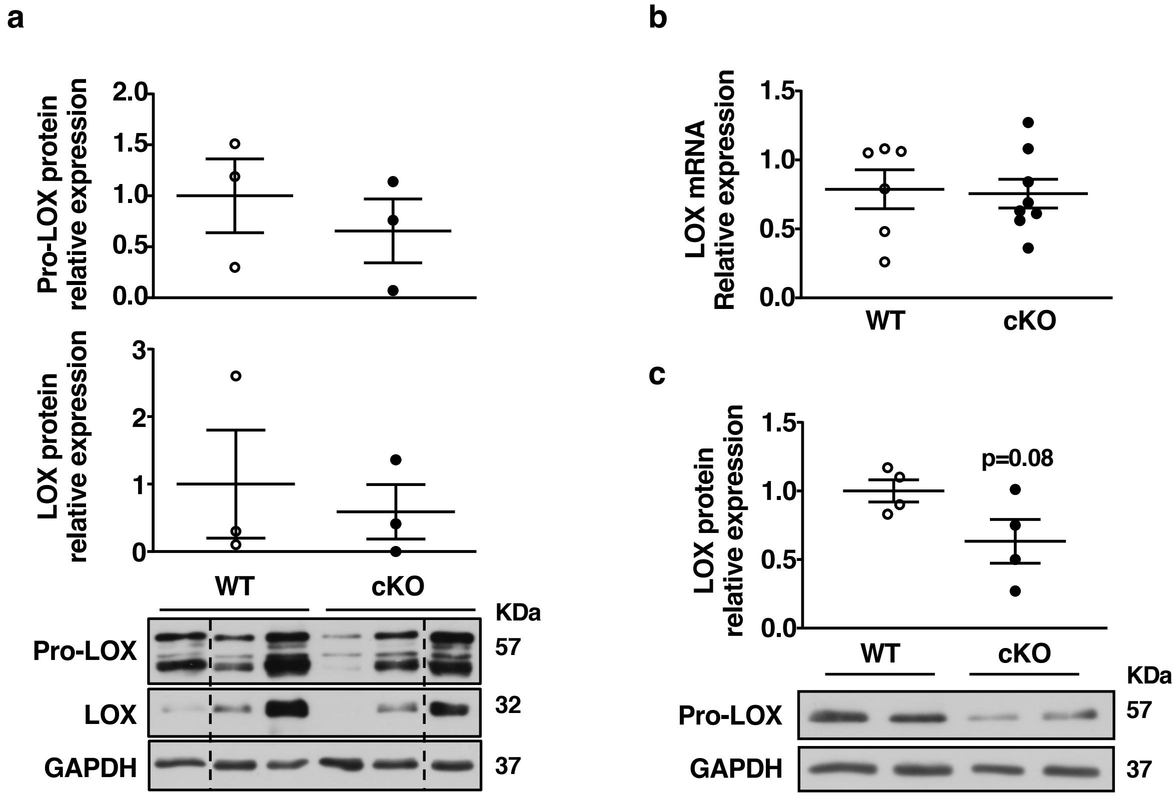

Supplementary Fig. 3 , related to Fig. 3 MF-specific AMPKα1 deletion does not impact lysyl oxidase expression in the infarcted myocardium **a** Western blot analysis of pro-lysyl oxidase (LOX) and LOX expression in the infarct area of MF-specific AMPKα1 KO (cKO) and littermate WT (WT) hearts 14 days after myocardial infarction (MI) (n=3 mice/group). **b** qRT-PCR analysis of LOX expression in the infarcted area of cKO and WT hearts 7 days after MI (n=6-8 mice/group). Results are expressed as fold change over WT. **c** CFs were isolated from cKO and WT hearts 14 days after MI and western blotting was performed to analyze pro-LOX expression (n=4 mice/group). (a-c) Dot-plots represent data from individual mice, as well as mean ± SEM. Statistical significance was determined using the unpaired t-test.

Supplementary Fig. 4

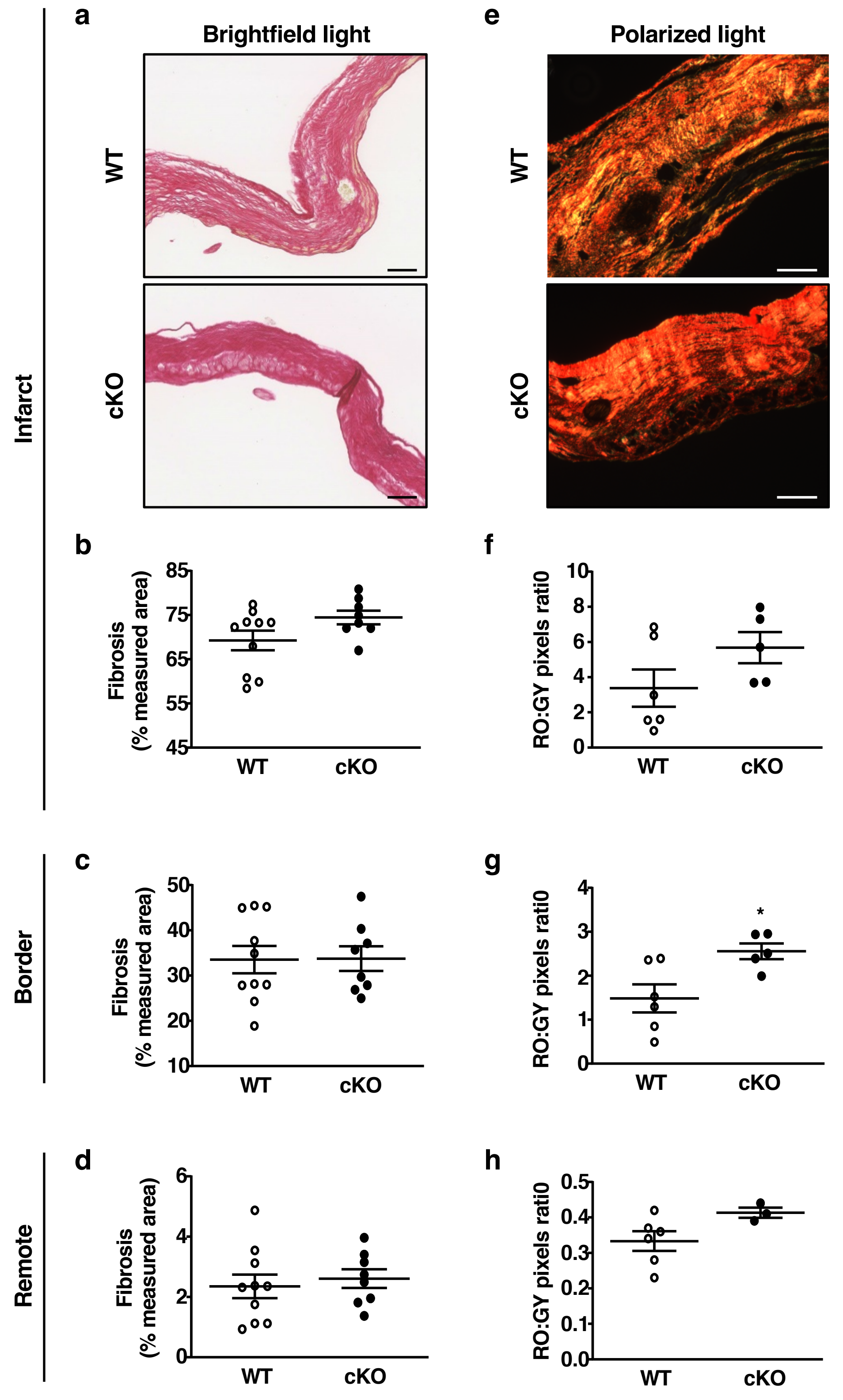

Supplementary Fig. 4, related to Fig. 3 MF-specific AMPKα1 deletion enhanced the kinetics of fibrotic response **a** Representative images of MF-specific AMPKα1 KO (cKO) and littermates WT (WT) infarct areas stained by picrosirius red and visualized under brightfield light, 3 months after myocardial infarction. Magnification: 10x. Scale bars: 100µm. **b-d** Quantifications of fibrosis in the infarct (b), border (c) and remote (d) areas of cKO and WT mice (n=8-10 mice/group). Results are expressed as % of measured tissue area. **e** Representative images of cKO and WT infarct areas stained by picrosirius red and visualized under polarized light. Magnification: 10x. Scale bars: 100µm. **f-h** Quantification of picrosirius red polarized light analysis in the infarct (f), border (g) and remote (h) areas of cKO and WT mice (n=5-6 mice/group). Results are expressed as red-orange on green-yellow pixel ratio in the measured tissue area. (a-h) Hearts were excised 3 months post-MI. Dot-plots represent data from individual mice, as well as mean ± SEM. Statistical significance was determined using unpaired t-test (c, d, f-h) or Mann-Whitney test (b). *p<0.05 compared to WT.

Supplementary Fig. 5

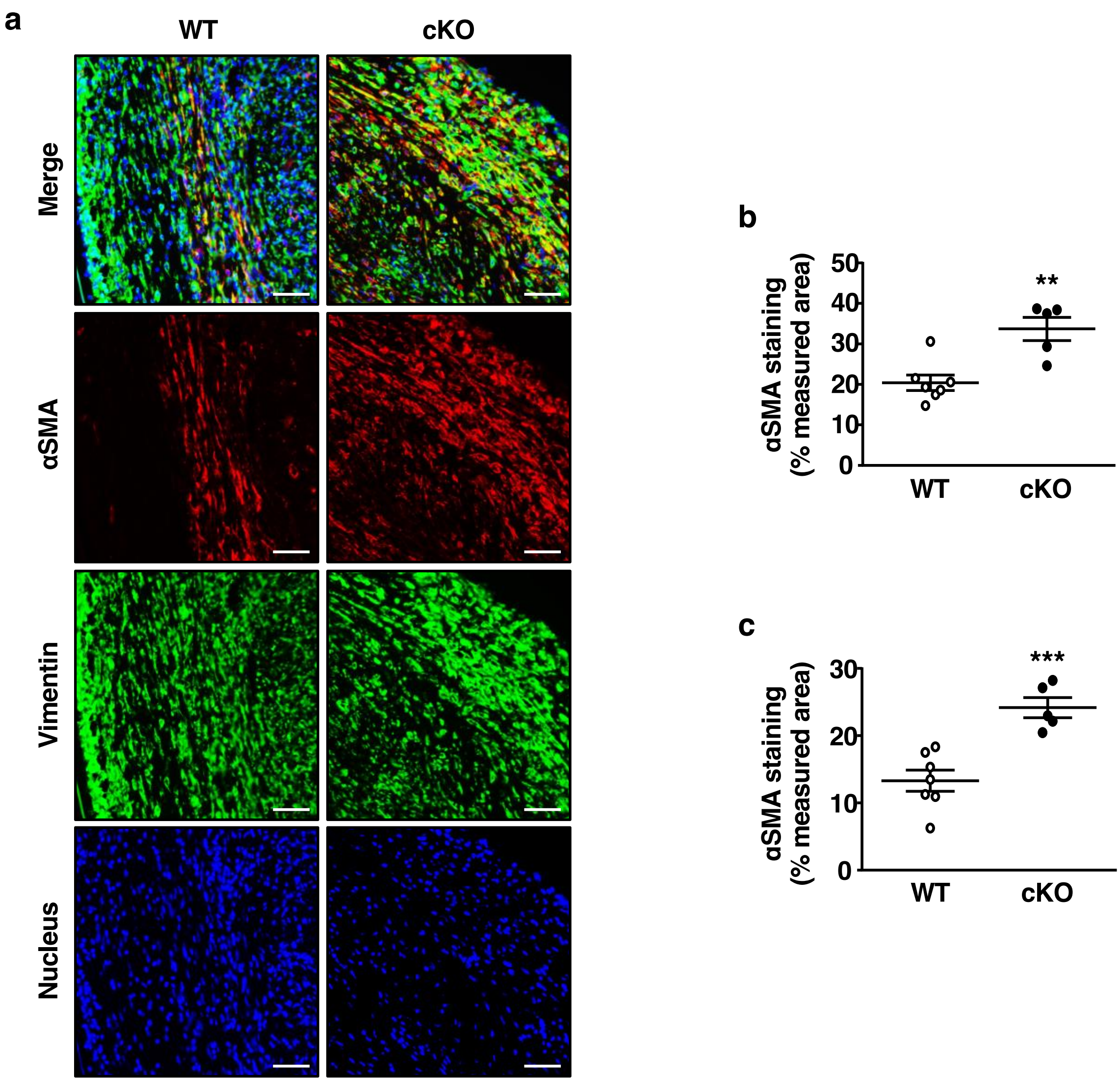

Supplementary Fig. 5, related to Fig. 4 MF-specific AMPKα1 deficiency promotes CF activity **a** Representative images of co-immunofluorescent staining of myofibroblasts (αSMA-positive: red fluorescence; vimentin-positive: green fluorescence) in the infarct area of MF-specific AMPKα1 KO (cKO) and littermates WT (WT) at 14 days post-myocardial infarction (MI). **b and c** Quantification of myofibroblasts in the infarct (b) or border (c) areas of cKO and WT (n=5-7 mice/group). Results are expressed as % of αSMA-stained area within the vimentin-stained tissue area. Magnification: 20x. Scale bars: 50μm. Dot-plots represent data from individual mice, as well as mean ± SEM. Statistical significance was determined by unpaired t-test. **p<0.01, ***p<0.001 compared to WT.

Supplementary Fig. 6

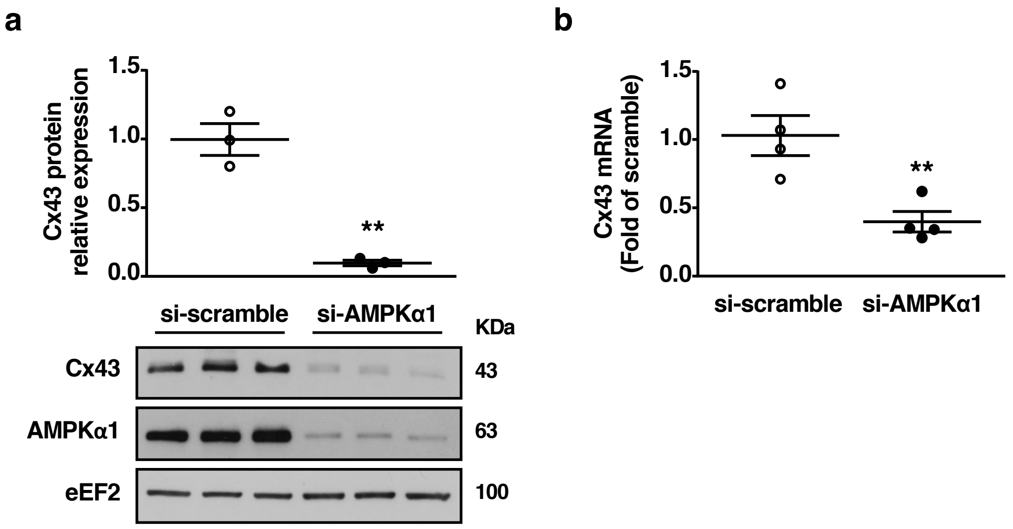

Supplementary Fig. 6, related to Fig. 6 Deletion of AMPKα1 in HEK293 suppresses Cx43 expression, as observed in HCFs **a-b** HEK293 were transfected with AMPKα1-targeting siRNA or scramble for 48 hours and Cx43 expression was assessed by western blotting (a) or qRT-PCR (b) (n=3 biological replicates/group). Dot-plots represent data from individual biological replicates, as well as mean ± SEM. Statistical significance was determined using unpaired t-test. **$p<0.01$ compared to si-scramble.

Supplementary Fig. 7

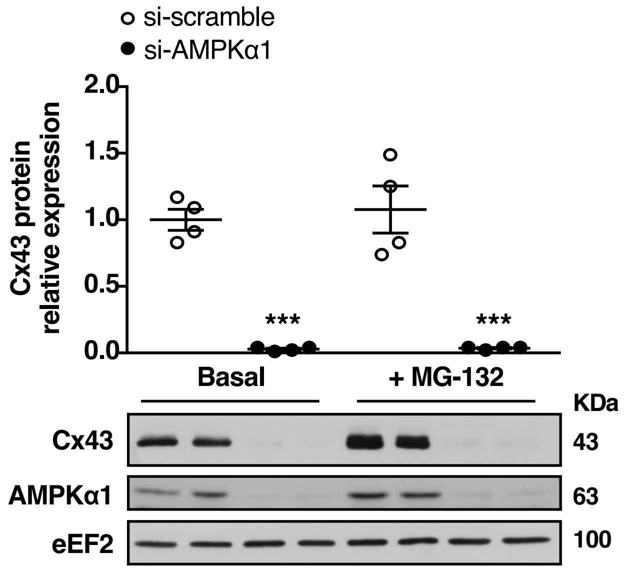

Supplementary Fig. 7, related to Fig. 6 AMPKα1 does not regulate Cx43 expression in CFs by modulating the proteasome Human CFs (HCFs) were transfected with AMPKα1-targeting siRNA or scramble for 48 hours and treated for 6 hours with MG-132. Cx43 expression was assessed by western blotting (n=4 biological replicates/group). Dot-plots represent data from biological replicates, as well as mean ± SEM. Statistical significance was determined using the two-way ANOVA followed by Sidak's multiple comparisons test. ***p<0.001 compared to corresponding si-scramble.
